# Phylogeny and biogeography of the wingless orthopteran family Rhaphidophoridae

**DOI:** 10.1038/s42003-024-06068-x

**Published:** 2024-04-02

**Authors:** Do-Yoon Kim, Sangil Kim, Hojun Song, Seunggwan Shin

**Affiliations:** 1 School of Biological Sciences, Seoul National University, Seoul, 08826 Republic of Korea; 2 Comparative Medicine Disease Research Center, Seoul National University, Seoul, 08826 Republic of Korea; 3 Research Institute of Basic Sciences, Seoul National University, Seoul, 08826 Republic of Korea; 4 Museum of Comparative Zoology and Department of Organismic and Evolutionary Biology, Harvard University, Cambridge, MA 02138 USA; 5 Department of Entomology, Texas A&M University, College Station, TX USA

**Keywords:** Entomology, Phylogenetics, Biogeography

## Abstract

Cave crickets (Rhaphidophoridae) are insects of an ancient and wingless lineage within Orthoptera that are distributed worldwide except in Antarctica, and each subfamily has a high level of endemicity. Here, we show the comprehensive phylogeny of cave crickets using multi-gene datasets from mitochondrial and nuclear loci, including all extant subfamilies for the first time. We reveal phylogenetic relationships between subfamilies, including the sister relationship between Anoplophilinae and Gammarotettiginae, based on which we suggest new synapomorphies. Through biogeographic analyses based on divergence time estimations and ancestral range reconstruction, we propose novel hypotheses regarding the biogeographic history of cave crickets. We suggest that Gammarotettiginae in California originated from the Asian lineage when Asia and the Americas were connected by the Bering land bridge, and the opening of the western interior seaway affected the division of Ceuthophilinae from Tropidischiinae in North America. We estimate that Rhaphidophoridae originated at 138 Mya throughout Pangea. We further hypothesize that the loss of wings in Rhaphidophoridae could be the result of their adaptation to low temperatures in the Mesozoic era.

## Abstract (web summary)

A new phylogenetic tree of cave crickets reveals relationships of all major lineages and suggests new hypotheses about their biogeographic history and origin.

## Introduction

Rhaphidophoridae (Orthoptera: Ensifera), commonly known as cave crickets, cave wētā, land shrimp, sand treaders, jumping, and camel crickets, are a wingless family consisting of nine extant subfamilies and one extinct subfamily with more than 1,100 described species^1^. They are considered the earliest diverging lineage of six families in the infraorder Tettigoniidea, one of two major lineages within Ensifera^2,3^. These insects are usually found in caves, burrows, cellars, and under logs, preferring dark and humid environments, and are characterized by their long legs and antennae, lack of wings, and often humped back^4,5^. Unlike most relatives in Ensifera, Rhaphidophoridae have no stridulatory and auditory organs for acoustic communication, but some species are known to produce courtship signals by tapping the abdomen or vibrating the body^6^. Interestingly, some species have been observed to visit flowers as potential plant pollinators in subarctic islands^7^.

**Fig. 1:**
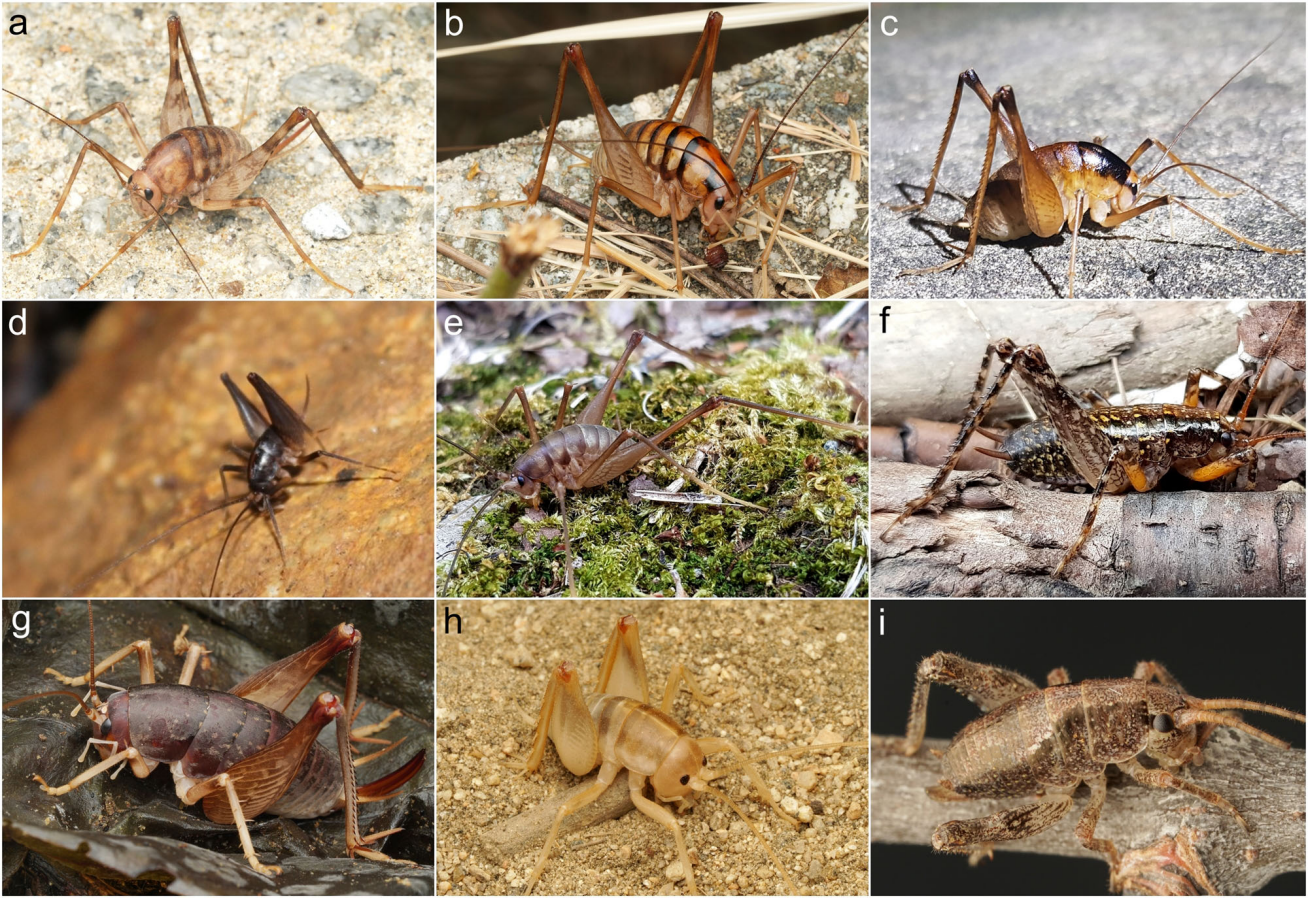
Representatives of the Asian and the American Rhaphidophoridae. **a** Aemodogryllinae, *Tachycines* (*Tachycines*) *asynamorus* Adelung (Taean, Korea). **b** Aemodogryllinae, *Tachycines* (*Tachycines*) *coreana* (Yamasaki) **stat. resurr**. (Jindo, Korea). **c** Aemodogryllinae, *Diestrammena* (*Diestrammena*) *unicolor* Brunner-Wattenwyl (Ulsan, Korea). **d** Aemodogryllinae, *Paratachycines* (*Paratachycines*) *ussuriensis* Storozhenko (Gwangju, Korea). **e** Aemodogryllinae, *Paratachycines* (*Hemitachycines*) *boldyrevi* (Uvarov) (Mt. Jirisan, Korea). **f** Anoplophilinae, *Anoplophilus koreanus* Storozhenko & Paik (Mt. Jirisan, Korea). **g** Rhaphidophorinae, *Rhaphidophora taiwana* Shiraki, 1930 (Ishigaki, Japan). **h** Ceuthophilinae, *Ceuthophilus* sp. (California, U.S.). **i** Gammarotettiginae, *Gammarotettix genitalis* Caudell (California, U.S.). Photographs by Do-yoon Kim.

Rhaphidophoridae are widely distributed across all continents except Antarctica, with each subfamily showing a geographically limited distribution^1^. For example, Aemodogryllinae and Rhaphidophorinae are distributed in Southeast Asia, with the latter family more widely distributed to the south, including Oceania (Fig. 1a–e, g). Anoplophilinae are distributed only in Far East Asia (Fig. 1f). Ceuthophilinae are widely distributed across North America (Fig. 1h), while the distribution of Tropidischinae and Gammarotettiginae is restricted to the west coast of North America (Fig. 1i). Dolichopodainae and Troglophilinae are distributed throughout the Mediterranean Sea. Macropathinae show a Gondwanian pattern and are distributed in South America, South Africa, Australia, Tasmania, and New Zealand. An extinct fossil subfamily, †Protroglophilinae, is found only in Baltic amber, which is estimated to have occurred 44 Mya in the Eocene epoch^8–10^. Because Rhaphidophoridae are primitively wingless, which potentially limits their ability to disperse and colonize, it is reasonable to hypothesize that geological events could have significantly impacted lineage diversification, resulting in their current distribution. Previous studies on cave-dwelling species showed that vicariant processes played an important role in shaping the complex paleogeographic histories of Dolichopodainae and Troglophilinae in the Mediterranean and of Macropathinae in the Southern Hemisphere^11–20^.

Numerous hypotheses on the origins of Rhaphidophoridae have been proposed based on the morphological or molecular phylogeny of related ensiferan groups^16,20–25^. Karny^26^ and Ander^21^ hypothesized that Rhaphidophoridae originated in the Southern Hemisphere and spread to the Northern Hemisphere, and Ander^21^ hypothesized that Dolichopodainae in the Mediterranean was the oldest representative subfamily. Hubbell and Norton^25^ stated that Macropathinae could be the subfamily most related to Ceuthophilinae in North America based on their morphological characteristics. However, Allegrucci et al.^16^ and Allegrucci and Sbordoni^20^ provided an alternative hypothesis based on molecular evidence, in which they proposed that Rhaphidophorinae and Aemodogryllinae in Southeast Asia would be the most closely related subfamilies to Macropathinae. In addition, they estimated the origin of Rhaphidophoridae in the Cretaceous period (117 Mya; 95% HPD: 105–130 Mya) and suggested that ancestors of Rhaphidophoridae must have been distributed in both the Southern and Northern Hemispheres since Pangaea. However, a recent phylogenomic study of Orthoptera^3^ led authors to estimate that the crown Tettigoniidea originated in the Permian (268 Mya; CI, 308.1–227.7 Mya) and Rhaphidophoridae in the Jurassic, both of which were considerably older estimates than those of previous studies.

Despite a long evolutionary history and a relatively large number of species, the morphological uniformity and lack of diagnostic characteristics within Rhaphidophoridae have made it difficult to robustly classify this family. For example, Anoplophilinae, which is found only in Far East Asia (Fig. 1a), has been a particularly enigmatic group. Ichikawa^27^ and Ishikawa^28^ first used the subfamily name Anoplophilinae, but it was rendered invalid due to the apparent lack of proper diagnosis and description. Otte^29^ considered each of the two genera of Anoplophilinae to be placed in two different extant subfamilies: the genus *Anoplophilus* Karny in the Mediterranean subfamily Troglophilinae and the genus *Alpinanoplophilus* Ishikawa in the North American Tropidischiinae. Kim and Kim^30^ also treated *Anoplophilus* as a member of Troglophilinae. On the other hand, Gorochov^31^ first proposed the species of Anoplophilinae to be closely related to the extinct subfamily †Protroglophilinae found in Baltic amber, followed by Ishikawa^32^ and Sugimoto and Ichikawa^33^, who placed the species of Anoplophilinae in †Protroglophilinae and considered them to be related to Ceuthophilinae in North America based on their morphological features. Later, Storozhenko and Paik^34^ established Anoplophilinae as a separate subfamily based on its morphological features, such as hind tibiae, a male subgenital plate, and ovipositor, but its relationships with other subfamilies remain unresolved. Despite these controversies, the systematics of the Asian Rhaphidophoridae have never been examined using molecular data.

In this study, we present the most comprehensive phylogeny of Rhaphidophoridae, in which all extant subfamilies, including the controversial subfamily Anoplophilinae, were sampled for the first time. The subfamilies Tropidischinae and Gammarotettiginae from western North America were also included for the first time. Based on our divergence time estimation and ancestral range reconstruction with time-stratified analyses, we propose new biogeographical hypotheses that are in line with the lineage diversification history within Rhaphidophoridae and major geological events. We also suggest a hypothesis regarding wing loss in Rhaphidophoridae, which will provide new insights into the origin and evolution of this interesting family.

## Results

### Phylogeny of rhaphidophoridae

**Fig. 2:**
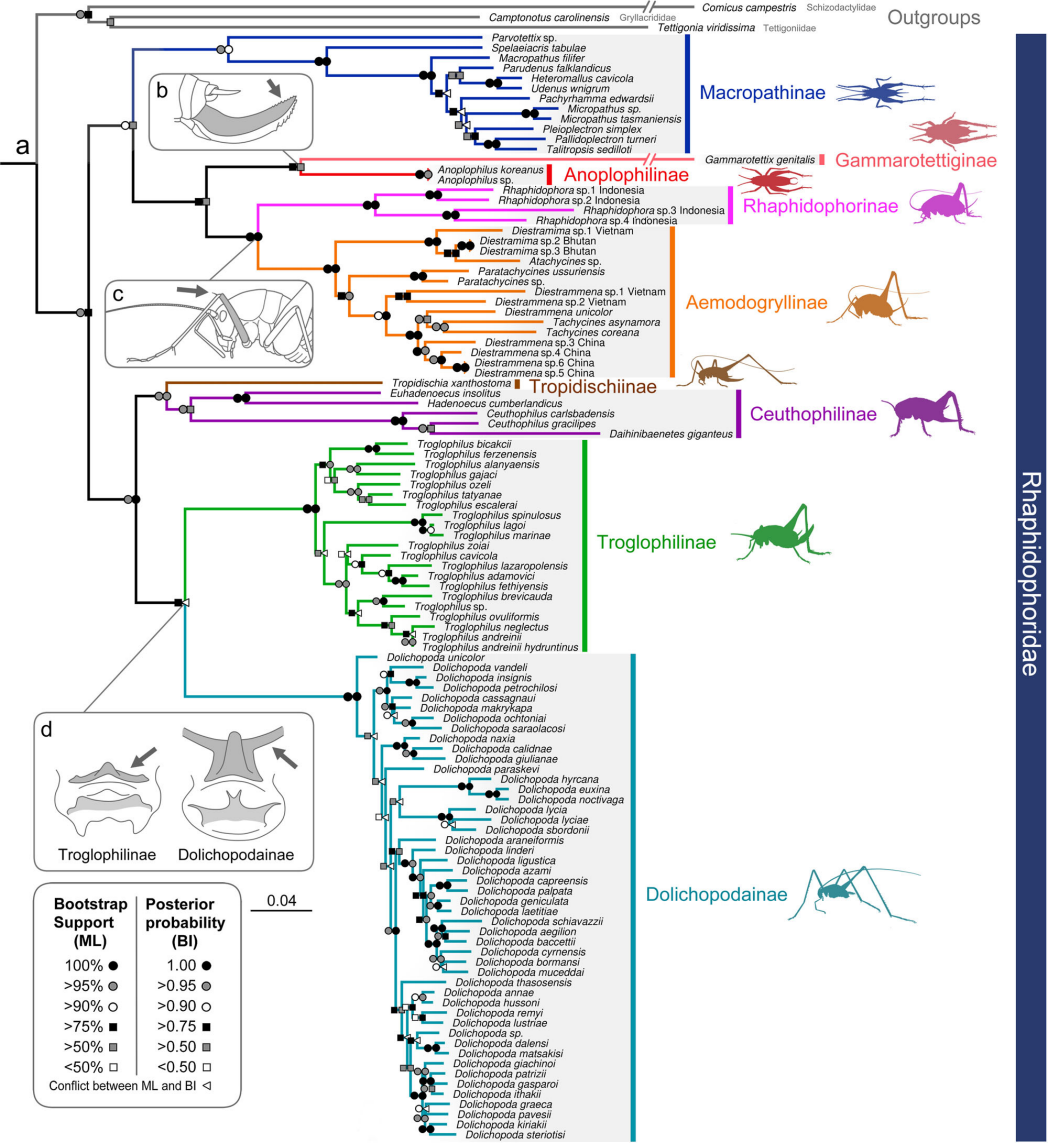
Molecular phylogeny of Rhaphidophoridae within the all extant subfamily and morphological synapomorphies supporting relationships. **a** The combined result of the phylogenetic analyses using 112 taxa and partitioned five multi-locus: the mitochondrial COI, 12 S rDNA, 16 S rDNA and the nuclear 18 S rDNA, 28 S rDNA. The topology is based on Maximum likelihood tree using IQ-tree with 2,000 bootstrap replications, and the bootstrap supporting values are indicated on the left of nodes. The result of Bayesian Inference using MrBayes are indicated by the posterior probabilities for nodal support values on the right of nodes. Topological differences between ML and BI trees are indicated by triangle marks on right of nodes. **b–d** Synapomorphy between the subfamilies is highlighted in grey, with significant anatomical structures pointed with arrows. **b** Denticles on upper valve of ovipositor. **c** An inner apical spine on fore femora. **d** Transversed epiphallic sclerite in male genitalia.

A total of 3,151 bp nucleotide sequences were used for phylogenetic reconstruction, including 951 bp of COI, 463 bp of 12 S rRNA, 590 bp of 16 S rRNA, 519 bp of 18 S rRNA, and 628 bp of 28 S rRNA. We recovered monophyletic Rhaphidophoridae in the dataset containing 112 species, with strong nodal support in both the ML and BI analyses (Fig. 2). All subfamilies were recovered as monophyletic except Tropidischiinae, which was a monotypic subfamily, and Gammarotettiginae, which included only one species in the analyzed dataset.

Rhaphidophoridae was largely divided into two lineages: (Macropathinae + ((Gammarotettiginae + Anoplophilinae) + (Rhaphidophorinae + Aemodogryllinae))) and ((Ceuthophilinae + Tropidischiinae) + (Dolichopodainae + Troglophilinae)). Macropathinae, which includes species from Australia, New Zealand, South Africa, and South America, was recovered as monophyletic, confirming a Gondwanan origin of the subfamily. Within Macropathinae, the species from South America and those from New Zealand each formed clades, whereas the species from Australia did not form a monophyletic group. The earliest diverging lineage within Macropathinae was *Parvotettix* sp. from Tasmania, and the next branching lineage was *Spelaeiacris tabulae* Péringuey in South Africa. However, internal relationships between the clades of species from South America, New Zealand, and several Australian species were incongruent between the BI and ML trees. In addition, the three Asian subfamilies did not form monophyletic groups. Rhaphidophorinae was found to be sister to Aemodogryllinae, but Anoplophilinae, whose phylogenetic placement had been previously unknown, was recovered as sister to Gammarotettiginae from the west coast of North America in both the ML and BI analyses. In Aemodogryllinae, the genus *Diestrammena* was recovered as paraphyletic because *Atachycines*, *Paratachycines*, and *Tachycines* were nested within *Diestrammena. Tachycines coreana* Yamasaki, which was previously synonymized under *T. asynamorus* Adelung, was separated from *T. asynamorus* as a distinct species with a relatively long branch length. Tropidischiinae was recovered as sister to Ceuthophilinae from North America. Troglophilinae was recovered as sister to Dolichopodainae from the Mediterranean region in the ML analysis, but in the BI analysis, it was recovered as sister to (Ceuthophilinae + Tropidischiinae). In Troglophilinae, the species of *Troglophilus* from insular Greece and Anatolia were recovered as monophyletic. *Gammarotettix genitalis* Caudell and *Comicus campestris* (one of the outgroups) each had a relatively long branch length in the ML tree.

### Divergence time estimate and biogeography

**Fig. 3:**
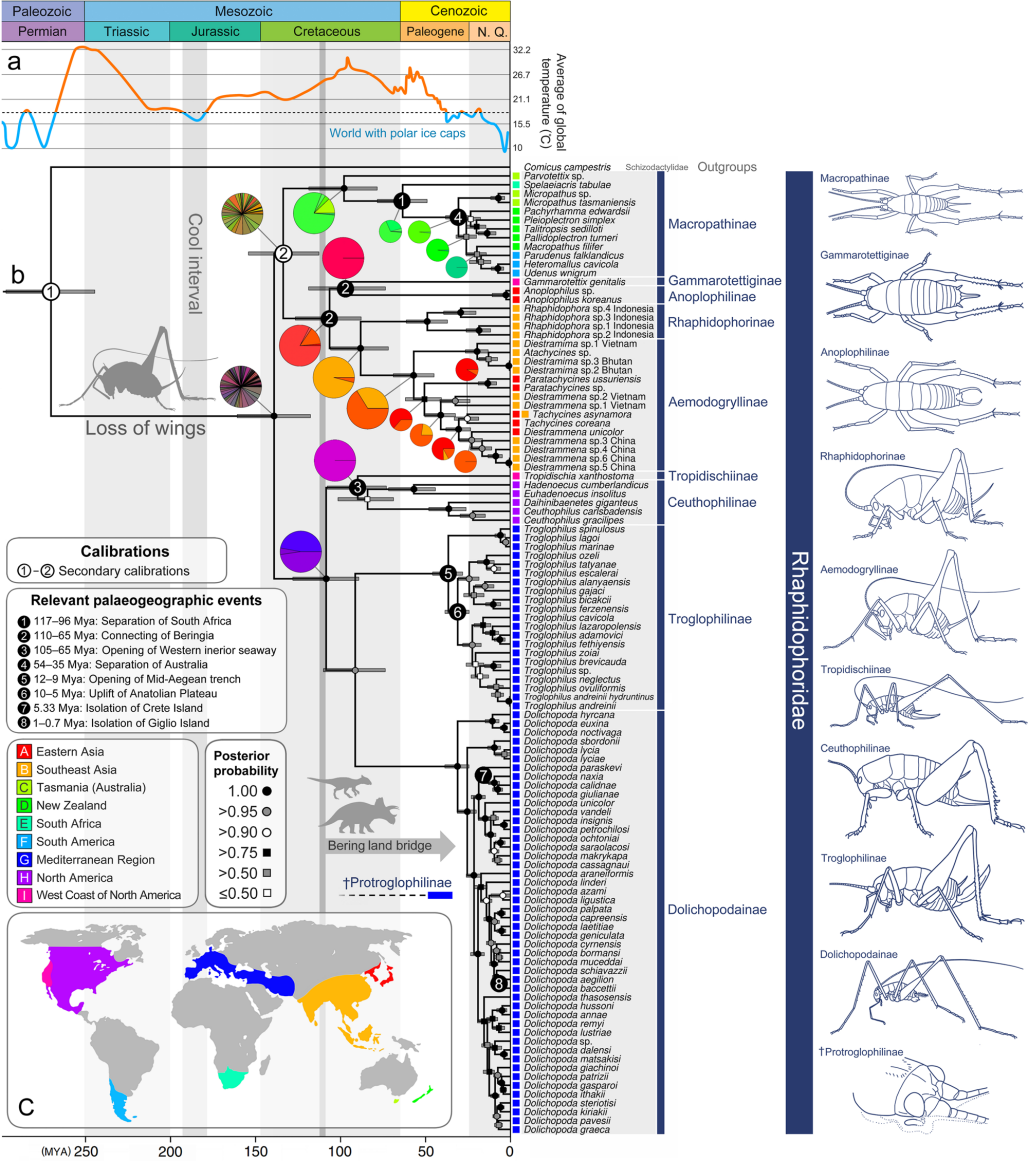
Dated phylogeny with biogeography and prehistoric events. **a** Average of global surface temperature, modified from preliminary results of a Smithsonian Institution project led by Scott Wing and Paul Huber (adapted from https://www.climate.gov/media/11332, accessed on 6 October 2022). **b** Estimated divergence time based on Bayesian Inference using MrBayes with Birth–death process for a tree model. The secondary calibration points are indicated by the white marks with black numbers on the nodes. Distribution informations of each species are labeled on the tips. The pie charts on nodes show the reconstructed ancestral state of distributions which are the results of biogeographic analyses using DIVALIKE + J model in BioGeoBEARS. The paleogeographic events that had been considered to be relevant lineage diversification of Rhaphidophoridae or suggested newly in this study are indicated by the black marks with white numbers on the nodes. The discussed prehistoric events are shown on the timeline. †Protroglophilinae, whose phylogenetic position has been unrevealed, is only marked according to the geological ages of the Baltic ambers. **c** Map for distribution of Rhaphidophoridae, referred from Cigliano et al. ^1^.

The estimated divergence time indicated that Rhaphidophoridae originated at approximately 138 Mya (early Cretaceous), and lineage diversification to subfamilies occurred during the Cretaceous period, radiating until the Cenozoic (Fig. 3). Both DIVALIKE + J and BAYAREALIKE + J were recommended as best-fit models in the BioGeoBEARS analysis with the same LnL scores (−45.92) (Figs. S2–S7; Table S4). The results of the two analyses were highly similar, and the result of DIVALIKE + J was selected for describing biogeographic events. The ancestral distribution of the most recent common ancestor (MRCA) of extant Rhaphidophoridae was proposed to various probabilities. All regions except for South America were believed to contribute to the ancestral distribution of the MRCA of extant Rhaphidophoridae around 138 Mya. The MRCA of Rhaphidophoridae was largely divided into two lineages: (Macropathinae + ((Anoplophilinae + Gammarotettiginae) + (Aemodogryllinae + Rhaphidophorinae))) lineage in Gondwana and Laurasia (Asia and West Coast of North America) and ((Ceuthophilinae + Tropidischiinae) + (Troglophilinae + Dolichopodainae)) lineage in western Laurasia (North America and the Mediterranean region).

Macropathinae shows the clearest distribution pattern that is characteristic of the ancient radiation in Gondwana, after having diverged at approximately 133 Mya. The ancestral distribution of MRCA of Macropathinae was proposed in Tasmania, New Zealand, and Africa. The earliest diverging lineage within this subfamily was *Parvotettix* species found in southern Australia (Tasmania), followed by a South African lineage, *Spelaeiacris tabulae*. The remaining species within the subfamily are closely related to each other, including those currently found in New Zealand and those found in South America.

The lineage distributed in Asia and California was proposed to have diverged from the common ancestor with Macropathinae to 139 Mya and recolonized to Laurasia (Asia and California). The Laurasian lineage was divided into the Anoplophilinae + Gammarotettiginae lineage in eastern Asia and the west coast of North America and the Rhaphidophorinae + Aemodogryllinae lineage in Southeast Asia at 106 Mya. Among them, Gammarotettiginae diverged from Anoplophilinae at 96 Mya in eastern Asia and the west coast of North America. In Southeast Asia, Aemodogryllinae diverged from Rhaphidophorinae. Within Aemodogryllinae, *Diestramima* Storozhenko + *Atachycines* Furukawa remained in Southeast Asia, whereas *Paratachycines* + *Diestrammena* + *Tachycine*s migrated to East Asia and diversified. Except *Paratachycines*, which first branched out in East Asia, *Diestrammena* and *Tachycines* had multiple dispersal events and recolonized Southeast Asia several times. The divergence time estimation without the constraint was different in detail, but the results of the biogeographic analyses consistently showed that Gammarotettiginae diverged in eastern Asia and the west coast of North America (Figs. S8–S13). In the unconstrained timetree, Gammarotettiginae was separated from the common ancestor of Anoplophilinae + Rhaphidophorinae + Aemodogryllinae at 122 Mya in Asia, including Beringia. The lineage that diverged from the MRCA of Rhaphidophoridae in western Laurasia was divided into two clades at 108 Mya: (Ceuthophilinae + Tropidischiinae) in North America and (Troglophilinae + Dolichopodainae) in the Mediterranean region. The Mediterranean lineage was further divided into Dolichopodainae and Troglophilinae at 91 mya. The extant species of Dolichopodainae and Troglophilinae were estimated to have radiated at 30 Mya and 36 Mya, respectively. Tropidischiinae was split from its common ancestor with Ceuthophilinae at 89 Mya and recolonized the west coast of North America. The west coast of North America was recolonized twice independently by Gammarotettiginae and Tropidischiinae, lineages with an ancient divergence history of 138 Mya.

## Discussion

This study represents the first molecular phylogenetic analysis that included all known subfamilies of Rhaphidophoridae. The monophyly of Rhaphidophoridae is supported based on molecular data. Our recovered topology was congruent with that described in previous studies^20^, and our results clarified the phylogenetic position of Anoplophilinae, Gammarotettiginae, and Tropidischiinae, which were previously unresolved. Our study also supports the establishment of Anoplophilinae as an independent subfamily^34^. As such, the previous hypotheses that placed the East Asian genus *Anoplophilus* in Troglophilinae in the Mediterranean^29,30^ are refuted based on our results. However, our study did not include the genus *Alpinoplophilus* in Anoplophilinae, which inhabits only Japan and Far East Russia, and therefore, the hypothesis that placed the genus *Alpinoplophilus* in Tropidischiinae in North America^29^ remains untested. In addition, the hypothesis that Anoplophilinae species were part of the extinct †Protroglophilinae found in Baltic amber^31–33^ could not be evaluated with molecular phylogeny. Unlike another hypothesis, which considered Gammarotettiginae to be sister to Ceuthophilinae^31^, our results revealed Gammarotettiginae as sister to Anoplophilinae. Although Gammarotettiginae was analyzed using one gene from public data, we were also able to present morphological evidence to support the relationship between the two groups. We propose the synapomorphies that unite Gammarotettiginae and Anoplophilinae as a monophyletic group include a straight dorsal profile and upper margin of the upper valve of the ovipositor with denticles at the apex (Fig. 2b). The denticles on the upper valve of the ovipositor of Anoplophilinae are weakly pronounced compared to those of Gammarotettiginae. The members of Gammarotettiginae, known as arboreal camel crickets, have tree-dwelling habitats^35^, and the members of Anoplophilinae are often found on trees (personal observation). This similarity in ecological behavior can also be considered a trait derived from the same ancestor, and the weakness of denticles on the upper valve of the ovipositor can be considered a result of the behavioral shift from arboreal to terrestrial habitats. Tropidischiinae, whose phylogenetic position was uncertain based on morphology alone^31^, has been found as sister to Ceuthophilinae from North America. However, we were unable to clearly define a synapomorphy that unites Tropidischiinae and Ceuthophilinae due to the wide range of morphological diversity in Ceuthophilinae. Macropathinae had been considered to be the earliest diverging lineage within Rhaphidophoridae^25,31^, but our study, as well as that by Allegrucci and Sbordoni^20^, refuted this hypothesis, as Rhaphidophoridae was found to be broadly divided into two major clades. Macropathinae is characterized by a few unique characters that were considered primitive by previous authors, but here, we consider them to be autapomorphic, such as the distal part of the male genital plate divided into upper and lower parts and the upper margin of the first segment of the hind tarsi with paired apical spines. Based on our phylogeny, we consider morphological synapomorphy that unites Rhaphidophorinae and Aemodogryllinae to be the inner apical spine on the fore femora (Fig. 2c). The ML tree showed Dolichopodainae and Troglophilinae to form a monophyletic group, which is supported by the transverse epiphallic sclerite in male genitalia (Fig. 2d).

**Fig. 4:**
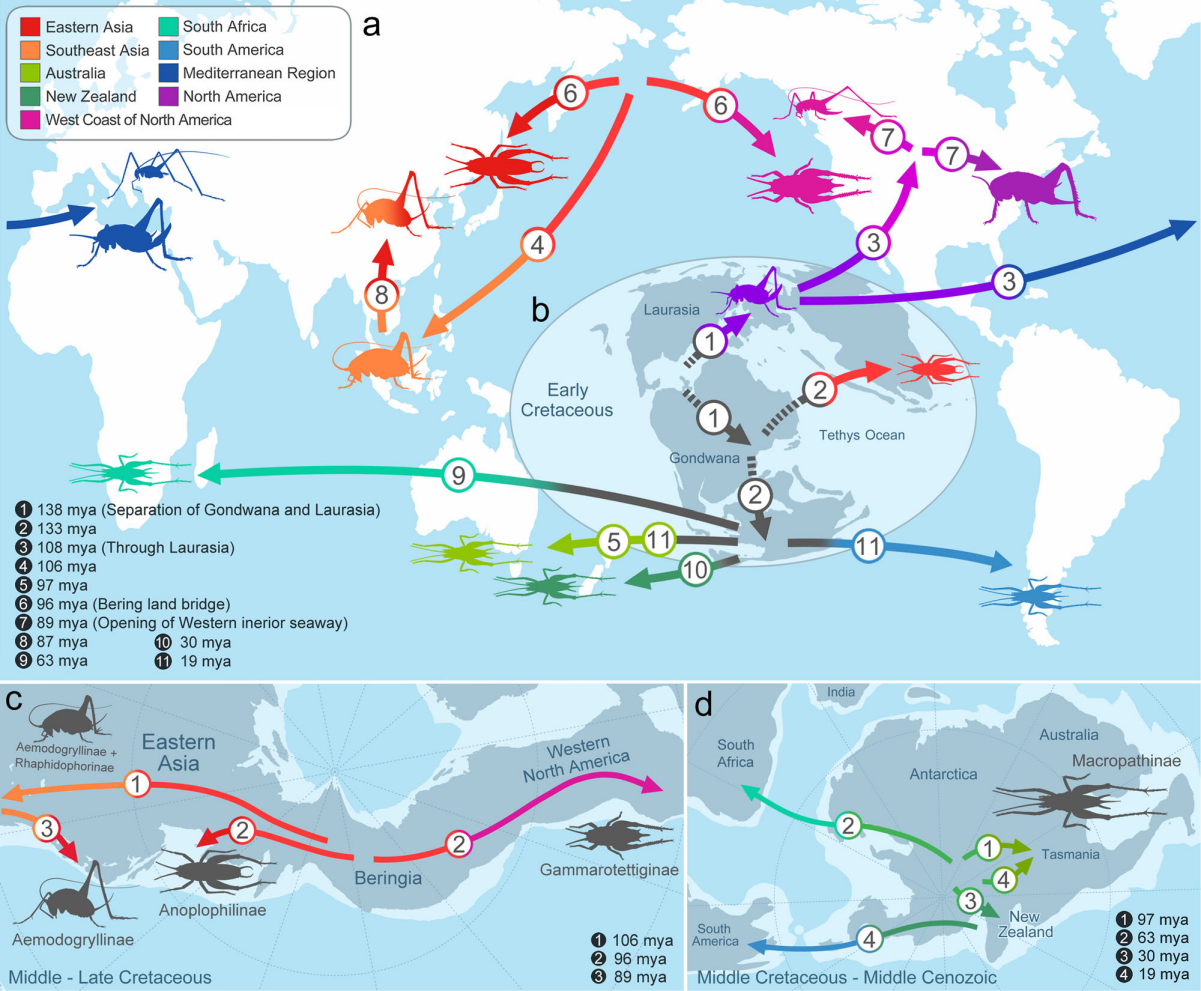
Historical biogeography scenario of Rhaphidophoridae. **a–b** Main dispersal events of extant lineages from 138 mya to the present day. **a** Map for present-day with the distributions of extant lineages. **b** Paleogeographic map for Early Cretaceous with the origin. **c** A dispersal event via Bering land bridge with origin in Eastern Asia. **d** Dispersal events of Macropathinae on the separating Gondwana. The numbering represents the colonization sequence proposed in the ancestral states reconstruction using BioGeoBEARS, and estimated divergence time with relevant palaeogeographic events are shown in the figure. Colour indicates the ancestral state of distributions and extant distributions. The dotted line at the start of the arrow represents reconstructed ancestral distribution which is ambiguous to show on the map. **b–d** Paleogeographic maps were modified from Scotese (2021).

Rhaphidophoridae, found on all continents except Antarctica, exhibit geographic endemism influenced by paleogeological events^11–20^. Using the taxonomically comprehensive dataset for the first time, we evaluated and reviewed the biogeographic hypothesis with estimated divergence time (Fig. 4). We found a new concurrence between our estimated divergence in Rhaphidophoridae and the age of specific paleogeological events, indicating that dispersal and vicariance occurred when the region was connected and separated in biogeographic history.

**Fig. 5:**
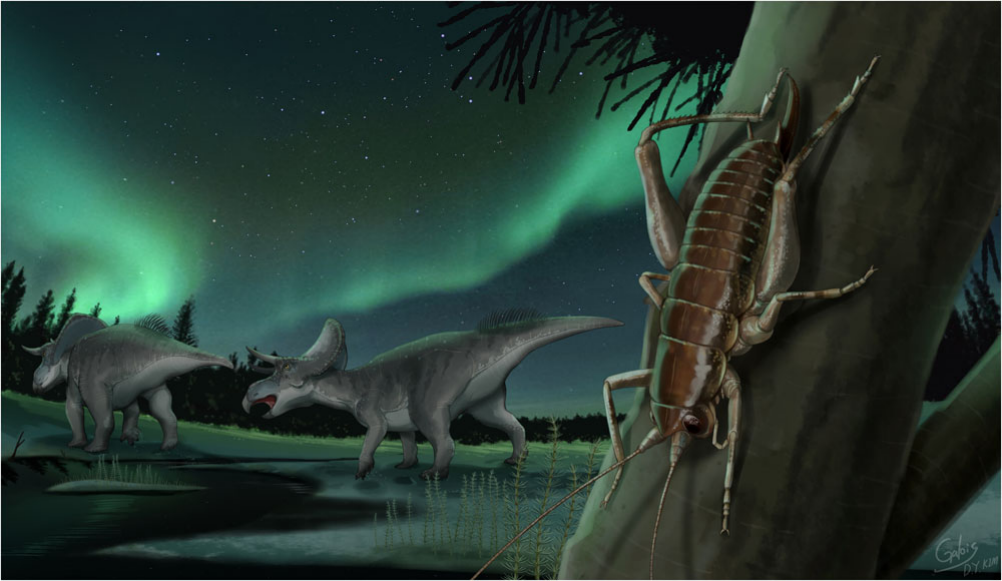
Artistic reconstruction of the Beringia in the Early Cretaceous. The descent of Asian and Californian lineage near the bering land bridge with ceratopsians (ancestor lineage of *Zuniceratops* Wolfe & Kirkland) (artwork by Do-Yoon Kim).

We propose a new biogeographic hypothesis suggesting that Gammarotettiginae in California originated near Beringia when Asia and America were connected by the Bering land bridge (Fig. 5). The connection between Asia and America by the Bering land bridge during the Cretaceous Period is supported by multiple trans-Beringia dispersals of dinosaurs such as ceratopsids, hadrosaurids and theropods (Fig. 4c)^36–39^. During the late Cretaceous Period, when Tropidischiinae diverged from Ceuthophilinae on the west coast of North America, the region was separated by the opening of the western interior seaway^40,41^. At 133 Mya, Macropathinae and the Asia-Beringian lineage were divided between Gondwana and eastern Laurasia, even though the two continents were already separated. However, fossil records suggest the possibility of a connection and exchange of biota between the two continents^42,43^. Since the distribution of the MRCA of Rhaphidophoridae was proposed to encompass all regions except South America in the ancestral state reconstruction, it is challenging to specify its exact origin. Our divergence time estimates sometimes deviate from previously considered paleogeological events. For instance, the divergence of *Spelaeiacris tabulae* from South Africa (63 Mya) postdates the separation of South Africa from Gondwana (117–96 Mya)^44,45^.

In our biogeographic analysis, we confirmed that some areas have been recolonized several times by various lineages. The west coast of North America was recolonized by both Gammarotettiginae from Beringia and Tropidischiinae, splitting from the common ancestor of the North American lineage. Southeast Asia also experienced multiple recolonizations by Rhaphidophorinae and some Aemodogryllinae lineages. The extant Mediterranean lineage radiated recently, and before this radiation, †Protroglophilinae, a fossil found in Baltic amber, could have occupied the ecological niche of the European region. The phylogenetic position of †Protroglophilinae remains unresolved, but based on the endemic distribution of Rhaphidophoridae, it could be hypothesized that †Protroglophilinae was closely related to western Laurasia lineages.

We inferred that the ancestral lineages that gave rise to Rhaphidophoridae could have been distributed throughout Pangaea around 138 Mya. The recent molecular phylogeny by Allegrucci and Sbordoni^20^ suggested a similar distribution in which Rhaphidophoridae originated in the Mesozoic Cretaceous period of 117 Mya, but our estimated age was older. The previous study, based on morphology, suggested the following existing hypothesis for the origin of Rhaphidophoridae: Ander^21^, who proposed Dolichopodainae as the oldest representative of Rhaphidophoridae; Hubbell and Norton^25^, who considered Macropathinae as an ancestor lineage with Ceuthophilinae; Gorochov^31^, who suggested Macropathinae as the most basal lineage of Rhaphidophoridae, and Ceuthophilinae and Gammarotettiginae is a direct descendant of Macropathinae, and Aemodogryllinae and Rhaphidophorinae sister to Dolichopodainae and Troglophilinae. Our study found that there is no single basal lineage, but the family consists of two major lineages that diverged early on.

Dispersal ability plays a key role in genetic differentiation to speciation^46,47^. In the same context, the endemic distribution and biogeographic history of Rhaphidophoridae are caused by their common trait, which is flightless with loss of wings. Although flightlessness and the loss of wings are common in Orthoptera^48–51^, Rhaphidophoridae is significantly older than other wingless orthopterans^3,52,53^. Flightlessness has long influenced their evolutionary history, and furthermore, the loss of wings in their common ancestor would imply the origin of Rhaphidophoridae. Therefore, we propose a narrative hypothesis about wing loss in Rhaphidophoridae, which could be a result of adaptation to low temperatures in the Mesozoic era. Although there are some exceptions^54^, the loss of wings in cold-specialized insects is common since they reduce their activity and metabolism for adaptation to low temperatures, and the frequency of encountering predators and flying away from them is low in cold environments^49,55^. Prior to the first divergence of the MRCA of Rhaphidophoridae, there was global climate cooling at the Middle–Late Jurassic transition (Late Callovian–Middle Oxfordian) (Fig. 3a)^56–59^. In addition, the dispersal path and distribution were restricted to near the polar regions (Fig. 4c and d) with relatively low temperatures. The extant cold-specialized insects, Boreidae (Mecoptera) and Grylloblattodea (Notoptera), were derived at a roughly similar age to Rhaphidophoridae, supported by fossil evidence^60,61^ and molecular estimation^62^. Moreover, extant Rhaphidophoridae species show a preference for low temperatures, such as alpine species that are found close to permanent ice^63^, subantarctic species^7^, and vast troglophile species, which can be the result of cold adaptation, relating to our hypothesis about the loss of wings. To support this hypothesis, further research is needed to precisely estimate divergence times and obtain genomic evidence of cold adaptation in wingless insects, including cave crickets.

## Conclusion

Our phylogenic analysis, which included all known subfamilies, revealed a unique and novel placement of the Asian subfamily Anoplophilinae. We also confirmed that the endemic distribution of Rhaphidophoridae as a result of winglessness is valid. Beringia, which connected Asia and North America, and the opening of the western interior seaway during the Cretaceous period coincide with the estimated divergence time of the Rhaphidophoridae lineages. The difference between the geological events and the molecular clock can be explained by several hypotheses regarding the dispersal capabilities of Rhaphidophoridae, but it can also be caused by a limitation of the dataset. Here, we suggest a hypothesis that global temperature and climate changes have affected lineage diversification and propose a narrative hypothesis that adaptation to low temperatures caused the loss of wings, leading to the endemic distribution of Rhaphidophoridae. Further research is needed to fully test these interesting hypotheses and gain a deeper understanding of the evolution and diversification of the cave cricket.

## Material and methods

### Taxon and character sampling

In total, 112 species from all nine extant subfamilies within Rhaphidophoridae were used for our ingroup taxon sampling process. We sampled one species of Anoplophilinae, represented by *Anoplophilus koreanus* Storozhenko and Paik, collected from Korea, and five species of Aemodogryllinae, collected from Korea and Russia. For Aemodogryllinae, we included Asian representatives of *Paratachycines* Storozhenko, *Tachycines* Adelung, and *Diestramena* Brunner von Wattenwyl. In addition, for this study, we incorporated sequence data from Tropidischinae and Gammarotettiginae and other species belonging to different subfamilies that were not previously analyzed in phylogenetic analyses. The sequence data for the remaining taxa, which were part of previous studies^14–20^ were retrieved from GenBank. For outgroups, we included three ensiferan species representing Schizodactylidae, Gryllacrididae, and Tettigoniidae, all of which belong to the infraorder Tettigoniidea (Table S1).

Genomic DNA was extracted from specimens’ legs preserved in 99% EtOH or dry-mounted using the DNeasy Blood and Tissue kit (QIAGEN, Inc.) according to the manufacturer’s guidelines. The voucher specimens were preserved in 100% ethanol and stored in a −80 °C deep freezer with matching extracted DNA in the School of Biological Sciences, Seoul National University, Seoul, South Korea (SNUE). Mitochondrial cytochrome *c* oxidase I (COI), small ribosomal subunit (12 S), large ribosomal subunit (16 S), and nuclear ribosomal RNA 18 S and 28 S gene fragments were selected for multigene phylogenetic analysis based on previous studies^14–20^. Fragments of the genes were amplified using AccuPower PCR primers (Bioneer, Korea), and information on the primers and PCR conditions for each gene is listed in Table S2. PCR products were visualized and confirmed by electrophoresis on a 2% agarose gel and sequenced using the Sanger method. The sequence data of each gene from both directions were assembled using SeqMan Pro v. 7.1.0 (DNASTAR, Inc., U.S.A).

### Molecular phylogenetic analyses

Protein-coding genes and ribosomal RNA genes were aligned using different methods. The sequence of mitochondrial COI was translated to its amino acid sequence for conservation of reading frames, aligned using MUSCLE^64^, and back-translated to nucleotides in MEGA X^65^. The ribosomal RNA sequences (12 S, 16 S, 18 S, and 28 S) were aligned using MAFFT ver.7^66^ with the E-INS-i method. All individual gene alignments were concatenated into a single matrix using FASconCAT-G^67^. The nucleotide substitution model for each gene partition was estimated using PartitionFinder 2^68^ with a greedy algorithm.

We performed maximum likelihood (ML) and Bayesian inference (BI) analyses on the concatenated dataset using a cluster computer on SNUE, and both analyses were performed by applying the recommended substitution model for each partition by PartitionFinder2. The ML analysis was performed using IQ-tree 1.6.2^69^ with 2,000 bootstrap replications. BI analysis was performed using MrBayes 3.2.6^70^. Except for the applied substitution models for each partition followed by PartitionFinder2, default priors were used for all other parameters. The posterior distribution was estimated using Markov chain Monte Carlo (MCMC) with four chains for 100 million generations and sampling every 5,000 generations. Using Tracer 1.7^71^, we determined that convergence of the run and all effective sample sizes (ESSs) of parameters were over 200. The trees were summarized in MrBayes 3.2.6^70^ by discarding the first 25% of the results as burn-in. In summarizing trees, the contype was set to ‘Allcompat’, which adds all compatible groups to the tree, and the minimum probability of partitions was assigned a value of 10.

### Divergence time estimation

The divergence time of Rhaphidophoridae was estimated using MrBayes 3.2.6^70^ in a Bayesian framework. Because this program requires only one outgroup, we kept *Comicus campestris* Irish, and two other outgroup taxa were removed from the dataset. The nucleotide substitution models proposed by PartitionFinder 2 were applied to each gene. Since a recent study showed that the birth-death prior produced stable results on molecular dating across all scenarios^72^, we used the birth–death process for a tree model. The posterior distribution was estimated using MCMC with four chains for 100 million generations and sampling every 10,000 generations. The process of examining the results and summarizing trees was performed with the same methods as the BI analysis using Tracer 1.7^71^ and MrBayes 3.2.6^70^. It was confirmed that all ESSs of the model parameters exceeded 200. A topological constraint was applied to the clade between Anoplophilinae and Gammarotettiginae for consistency in topologies of the molecular clock dating tree with the ML tree and BI tree. Although fossil records provide reliable calibrations for molecular clock and lineage diversification, we were unable to use fossil calibrations in this study. †Protroglophilinae, the extinct Rhaphidophoridae subfamily found in Baltic amber, could not be used for calibration since its phylogenetic position was ambiguous. Gorochov^31^ proposed the phylogenetic position of †Protroglophilinae, but his proposal was challenging to confirm with recent molecular phylogenetic studies^16,20^ and our own phylogenetic analysis due to differing relationships between the subfamilies. In addition, while previous studies have utilized paleogeographic events to calibrate their time tree^20^, we opted not to use such events for calibration to re-evaluate biogeographic hypotheses based on the divergence time estimated by only molecular clocks. Instead, we relied on divergence time estimates from the most recent phylogenomic study^3^ of Orthoptera as our secondary calibration points. In this study, we used two calibration points: (1) 268 Mya as the origin of crown-Tettigoniidea, at the base of the phylogeny, and (2) 138 Mya, at the node where there was a division of Macropathinae and Aemodogryllinae + Rhaphidophorinae + Anoplophilinae + Gammarotettiginae.

### Ancestral range estimation for biogeographic analyses

The biogeographical history of Rhaphidophoridae was analyzed using the package BioGeoBEARS in R 4.1.0^73^. Based on the divergence time estimation, ancestral range estimation was performed excluding an outgroup. We defined the distribution of Rhaphidophoridae in nine areas: West Coast of North America (California), North America, Mediterranean Region, South America (including Falkland Islands), South Africa, Tasmania (Australia), New Zealand, Eastern Asia (Korea, Japan, and Far East Russia), and Southeast Asia (South China, Vietnam, Bhutan, Indonesia, and Philippines). The distribution of taxa was obtained from previous studies that retrieved data^19,20^ or the Orthoptera Species File^1^. A time-stratified analysis with dispersal probabilities specified for each period was conducted to consider the junction and separation of biographic areas according to continental drift during the diversification time of Rhaphidophoridae, which is nearly 150 Mya. The time scale was stratified into 30 million-year slices^74^: 0–30 Mya, 30–60 Mya, 60–90 Mya, 90–120 Mya, and 120–150 Mya (Fig. S1). The dispersal probabilities were scored by the following categories according to the connectivity of the biogeographic areas^74,75^: 0.01 for well-separated areas by water, 0.1 for moderately separated or connected areas, but the other area was inserted between the areas, or the areas were distant and separated by two or more land masses, and 1.0 for contiguous areas (Table S3). The connectivity of the biogeographic area over time was determined according to Scotese^76^.

We used six biogeography models: (1) DEC (dispersal-extinction-cladogenesis)^77^; (2) DEC + J (including founder-event speciation); (3) DIVALIKE, a likelihood version of DIVA (dispersal-vicariance)^78^; (4) DIVALIKE + J (including founder-event speciation); (5) BAYAREALIKE, a likelihood version of BayArea (Bayesian inference of historical biogeography for discrete areas)^79^; and (6) BAYAREALIKE + J (including founder-event speciation). Two parameters, d = dispersal and e = extinction, were included in these six models. We compared the likelihood values of the models using the likelihood ratio test, and the most likely model was selected using the Akaike information criterion (AIC)^80,81^.

### Reporting summary

Further information on research design is available in the Nature Portfolio Reporting Summary linked to this article.

## Supplementary information

Supplementary Information

Reporting Summary

## Data Availability

The additional supporting information of this study are available in the supplementary material of this article. Datasets are archived at the Zenodo Digital Repository at 10.5281/zenodo.8026258.
