# Supplementary material for: Phylogeny and biogeography of the wingless orthopteran family Rhaphidophoridae
Source: Commun Biol. 2024 Apr 2;7:401. doi: 10.1038/s42003-024-06068-x (PMC10987581; doi:10.1038/s42003-024-06068-x)
Supplement: Supplementary file 1 — Supplementary Information [file 42003_2024_6068_MOESM1_ESM.pdf]

**Supplementary Information**

# Phylogeny and biogeography of the wingless orthopteran family Rhaphidophoridae

Do-Yoon Kim, Sangil Kim, Hojun Song, and Seunggwan Shin

**Table of Contents**

A. Supplementary Tables
B. Supplementary Figures
C. Supplementary Note
D. Supplementary References

## A. Supplementary Tables

**Table S1.** Rhaphidophoridae and outgroups taxa with Genbank accession numbers of individual loci included in this study.

| Species | Collection localities | COI | 12S | 16S | 18S | 28S |
|---|---|---|---|---|---|---|
| **Outgroups** | | | | | | |
| *Tettigonia viridissima* | N/A | KT358272 | Z97606 | Z97622 | Z97587 | EU203932 |
| *Camptonotus carolinensis* | N/A | complete mitochondrion genome: KM657333 | | | KF570818 | KF570941 |
| *Comicus campestris* | N/A | complete mitochondrion genome: KM657337 | | | KM853292 | KM853397 |
| **Macropathinae** | | | | | | |
| *Micropathus tasmaniensis* | Slug Inn Cave (IB-152), Ida Bay, Tasmania | N/A | HM594537 | HM594505 | MK993682 | HM594569 |
| *Micropathus* sp. | Unnamed cave, Gordon-Albert karst area, Tasmania | N/A | MH171950 | MH171970 | MH171988 | MH172006 |
| *Pachyrhamma edwardsii* | Mountains Kaukau, Wellington, New Zealand | N/A | HM594541 | HM594509 | MK993684 | HM594573 |
| *Pleioplectron simplex* | Caversham, Dunedin, New Zealand | N/A | HM594544 | HM594512 | HM594480 | HM594576 |
| *Talitropsis sedilotti* | Papaitonga, Levin, New Zealand | JN409935 | HM594543 | HM594511 | HM594479 | HM594575 |
| *Pallidoplectron turneri* | Waitomo, New Zealand | N/A | HM594545 | HM594513 | HM594481 | HM594577 |
| *Macropathus filifer* | Te Anaroa, Golden Bay, New Zealand | N/A | HM594542 | HM594510 | HM594478 | HM594574 |
| *Parudenus falklandicus* | Falkland Islands | N/A | HM594534 | HM594502 | MK993685 | HM594566 |
| *Heteromallus cavicola* | Milodon Cave, Puerto Natales, Chile | N/A | HM594531 | HM594499 | MK993686 | HM594563 |
| *Udenus w-nigrum* | Mount Aymond, Pali Aike National Park, Chile | N/A | HM594536 | HM594504 | MK993690 | HM594568 |
| *Spelaeiacris tabulae* | Bat's Cave, Cape Town, South Africa | N/A | HM594525 | HM594493 | MK993691 | HM594557 |
| *Parvotettix* sp. | Gunns Plains Cave, Gunns Plains, Tasmania | N/A | MH171945 | MH171965 | N/A | MH172003 |
| **Anoplophilinae** | | | | | | |
| *Anoplophilus koreanus* | Jirisan Mt., Gyeongsangnam-do, Korea | OQ779577 | OQ756226 | OQ779538 | OQ779552 | OQ779546 |
| *Anoplophilus* sp. | Kii Peninsula, Japan | N/A | N/A | N/A | AB647229 | N/A |
| **Aemodogryllinae** | | | | | | |
| *Atachycines* sp. | Cerrapunji cave, Khasi Hills, Meghalaya, India | N/A | HM594556 | HM594524 | MK993694 | HM594588 |
| *Diestrammena* sp.5 China | Jing Long Dong (Gold Dragon Hole), Guangdong, QingXin County, China | N/A | HM594552 | HM594520 | MK993695 | HM594584 |
| *Diestrammena* sp.4 China | Guangdong, Ruyuan County, Ting Xia Wo Dang, China | N/A | HM594553 | HM594521 | MK993696 | HM594585 |

| | | | | | | |
|---|---|---|---|---|---|---|
| *Diestrammena* sp.5 China | Guangdong Artificial Cave, Yingde City, China | N/A | HM594554 | HM594522 | MK993697 | HM594586 |
| *Diestrammena* sp.6 China | Shui Chi Dong (Water Pool Cave), Bei Pan Jiang, Zhen Feng County, Guizhou Province, China | N/A | HM594555 | HM594523 | MK993698 | HM594587 |
| *Diestrammena* sp.1 Vietnam | Marble Mountain Caves, Nha Trang, Vietnam | N/A | MK993664 | MK993652 | MK993673 | MK993658 |
| *Diestrammena* sp.2 Vietnam | Dang cave, Cuc Phuong National Park, Vietnam | N/A | MK993665 | MK993653 | MK993700 | MK993659 |
| *Diestrammena unicolor* | Baekunsan Mt., Gangwon-do, Korea | OQ779578 | OQ756229 | OQ779535 | OQ779547 | OQ779545 |
| *Tachycines asynamorus* | Yongmasan Mt., Gwangjin-gu, seoul, Korea | OQ779581 | OQ756227 | OQ779539 | OQ779551 | OQ779544 |
| *Tachycines coreana* | Noja Mt., Geoje Island, Korea | OQ779579 | OQ756228 | OQ779536 | OQ779548 | OQ779543 |
| *Paratachycines ussuriensis* | Noja Mt., Geoje Island, Korea | OQ772206 | OQ756230 | OQ779540 | OQ779550 | OQ779542 |
| *Paratachycines* sp. | Pokrovsky park, Vladivostok, Russia | OQ779580 | OQ756231 | OQ779537 | OQ779549 | OQ779541 |
| *Diestramima* sp.1 Vietnam | Cat Ba Islands, Hai Phong (epigean, in forest), Vietnam | N/A | MK993666 | MK993654 | MK993675 | MK993660 |
| *Diestramima* sp.2 Bhutan | Buyang-Chung Du, Tashi Yangse discrict, Bhutan | N/A | HM594551 | HM594519 | MK993703 | HM594583 |
| *Diestramima* sp.3 Bhutan | Dongdichu, Tashi Yangse discrict, Buthan | N/A | HM594550 | HM594518 | MK993702 | HM594582 |
| **Rhaphidophorinae** | | | | | | |
| *Rhaphidophora* sp.1 Indonesia | Gua Saripa (Saripa Cave), Bantimurung, Sulawesi, Indonesia | N/A | HM594548 | HM594516 | MK993704 | HM594580 |
| *Rhaphidophora* sp.2 Indonesia | Gua Londa (Londa Cave), Rantepao, Sulawesi, Indonesia | N/A | HM594549 | HM594517 | MK993705 | HM594581 |
| *Rhaphidophora* sp.3 Philippines | Urrak Cave, Mantalongon, Cebu Is., Philippines | N/A | HM594546 | HM594514 | MK993706 | HM594578 |
| *Rhaphidophora* sp.4 Philippines | Maitong Cave, Behind the clouds, Batuan, Bohol Is, Philippines | N/A | HM594547 | HM594515 | MK993707 | HM594579 |
| **Ceuthophilinae** | | | | | | |
| *Ceuthophilus gracilipes* | Hamden, CT, USA | KU376838 | MK993663 | AY793561 | MK993672 | MK993657 |
| *Ceuthophilus carlsbadensis* | USA | KU377018 | Z97597 | Z97613 | Z97563 | N/A |
| *Daihinibaenetes giganteus* | USA | KU889607 | N/A | AF212058 | N/A | N/A |
| *Euhadenoecus insolitus* | Indian Grave Point Cave, The Kalb Co., TN, USA | AY793591 | EF216948 | AY793563 | MK993679 | EF217005 |
| *Hadenoecus cumberlandicus* | Bat Cave, Carter Cave State Park, Carter Co., KY, USA | AY793592 | EF216947 | AY793562 | MK993680 | EF217004 |
| **Gammarotettiginae** | | | | | | |
| *Gammarotettix genitalis* | USA | N/A | N/A | N/A | N/A | KM853515 |
| **Tropidischiinae** | | | | | | |
| *Tropidischia xanthostoma* | USA | MG467592 | N/A | AF514480 | N/A | AF514436 |
| **Troglophilinae** | | | | | | |
| *Troglophilus cavicola* | Belon cave, Grone, Bergamo, North Italy | KY412391 | KY412201 | KY412248 | KY412295 | KY412341 |
| *Troglophilus neglectus* | Proteo cave, Sagrado, North Italy | complete mitochondrion genome: EU938374 | | | KF570820 | KF570948 |
| *Troglophilus andreinii* | Fico cave, Putignano, South Italy | KY412385 | KY412198 | KY412245 | KY412292 | KY412338 |
| *Troglophilus andreinii hydruntinus* | Diavoli Cave, Lecce, Apulia,South Italy | KY412386 | KY412199 | KY412246 | KY412293 | KY412339 |
| *Troglophilus* sp. | Ropa Mljet, Croatia | N/A | KY412206 | KY412253 | KY412300 | KY412346 |
| *Troglophilus ovuliformis* | Zavala Vjetrenica, Herzegovina | KY412395 | KY412208 | KY412255 | KY412302 | KY412348 |
| *Troglophilus brevicauda* | Pirro cave, Skrapar, Albania | KY412398 | KY412211 | KY412258 | KY412305 | KY412351 |
| *Troglophilus lazaropolensis* | Lazaropole, Macedonia | KY412394 | KY412207 | KY412254 | KY412301 | KY412347 |
| *Troglophilus zoiai* | Mayer's Cave, Stromi, Fokidos (Mt. Giona), Greece | KY412403 | KY412216 | KY412263 | KY412310 | KY412356 |
| *Troglophilus spinulosus* | Cave Spilaio Lera (Stavros), near Gouverneto, 11.29 km northeast of Chania, Crete, Greece | KY412410 | KY412225 | KY412272 | KY412318 | KY412365 |
| *Troglophilus lagoi* | Rhodos Island | KY412407 | KY412220 | KY412267 | KY412313 | KY412360 |
| *Troglophilus marinae* | Santorini Island | KY412408 | KY412221 | KY412268 | KY412314 | KY412361 |
| *Troglophilus ozeli* | Havran cave | KY412426 | KY412239 | KY412286 | KY412332 | KY412379 |
| *Troglophilus fethiyiensis* | Mugla Fethiye, Guroluk cave | KY412424 | KY412237 | KY412284 | KY412330 | KY412377 |

| | | | | | | |
|---|---|---|---|---|---|---|
| *Troglophilus adamovici* | Konya, Seydisehir, Tinatzepe cave | KY412419 | KY412232 | KY412279 | KY412325 | KY412372 |
| *Troglophilus bicakcii* | Konya, Derebucak,Bicacki cave | KY412430 | KY412240 | KY412287 | KY412333 | KY412380 |
| *Troglophilus ferzenensis* | Konya, Ferzene cave | KY412423 | KY412235 | KY412282 | KY412328 | KY412375 |
| *Troglophilus alanyaensis* | Alanya, Dim Cave | KY412414 | KY412227 | KY412274 | KY412320 | KY412367 |
| *Troglophilus gajaci* | Icel Silifke, Cennet cave | KY412421 | KY412234 | KY412281 | KY412327 | KY412374 |
| *Troglophilus escalerai* | Karaman- Maras, Dongel cave | KY412418 | KY412231 | KY412278 | KY412324 | KY412371 |
| *Troglophilus tatyanae* | Artvin, Kafkasor | KY412431 | KY412244 | KY412291 | KY412337 | KY412384 |
| **Dolichopodainae** | | | | | | |
| *Dolichopoda linderi* | Sirach Cave, Eastern Pyrenees | KY426936 | JF826039 | AY793567 | MK993708 | JF826061 |
| *Dolichopoda bormansi* | Brando Cave, Corsica Island | AY793627 | JF826047 | AY793578 | MK993709 | JF826069 |
| *Dolichopoda cyrnensis* | Valletto Cave, Corsica Island | AY793620 | JF826050 | AY793577 | MK993710 | JF826072 |
| *Dolichopoda azami* | Corno Cave, Piemonte | AY793604 | JF826040 | AY793568 | MK993711 | JF826062 |
| *Dolichopoda baccettii* | Punta degli Stretti Cave, Tuscany | AY793639 | JF826046 | AY793571 | MK993712 | JF826068 |
| *Dolichopoda aegilion* | Campese Mine, Giglio Island, Tuscany | AY793600 | JF826045 | AY793570 | MK993713 | JF826067 |
| *Dolichopoda schiavazzii* | Pipistrelli Cave, Tuscany | AY793633 | JF826044 | AY793573 | MK993714 | JF826066 |
| *Dolichopoda muceddai* | Limbara Mount, Sardinia | AY793629 | JF826051 | AY793575 | MK993715 | JF826073 |
| *Dolichopoda laetitiae* | Diavolo cave, Tuscany | KY426938 | JF826052 | AY793580 | MK993716 | JF826074 |
| *Dolichopoda geniculata* | Valmarino Cave, Latium | KY426937 | JF826055 | AY793583 | MK993717 | JF826077 |
| *Dolichopoda capreensis* | San Michele Cave, Capri Island, Campania | AY793606 | JF826059 | AY793587 | MK993718 | JF826081 |
| *Dolichopoda palpata* | Tremusa cave, Calabria | AY793608 | JF826060 | AY793588 | MK993719 | JF826082 |
| *Dolichopoda araneiformis* | Kod Solina Cave, Govedari, Miljet | EF217019 | EF216944 | EF216974 | MK993720 | EF216982 |
| *Dolichopoda remyi* | Waterfall Cave, Edessa, Pella | AY793637 | EF216939 | EF216969 | MK993721 | EF217001 |
| *Dolichopoda hussoni* | Apano Skala Cave, Naoussa, Imathia | EF2217032 | EF216943 | EF216973 | MK993722 | EF216990 |
| *Dolichopoda annae* | Small caves, Kato Olimpos, Leptokaria, Kalipefki | EU887891 | EU887845 | EU887860 | MK993723 | EU887875 |
| *Dolichopoda thasosensis* | Drakotripa Cave, Panayia, Thasos Island, Kavala | EF217020 | EF216926 | EF216956 | MK993724 | EF216983 |
| *Dolichopoda graeca* | Perama Cave, Ioannina, Epiro | EF217013 | EF216923 | EF216953 | MK993725 | EF216979 |
| *Dolichopoda steriotisi* | Antropograva Cave, Klimatia, Kerkira, Corfu | EF217016 | EF216925 | EF216955 | MK993726 | EF216981 |
| *Dolichopoda gasparoi* | Chirospilia Cave, Evghiros, Levkada | EF217008 | EF216920 | EF216950 | MK993727 | EF216976 |
| *Dolichopoda ithakii* | Marmarospilia cave, Vathi, Ithaki Island | EF217006 | EF216919 | EF216949 | MK993728 | EF216975 |
| *Dolichopoda patrizii* | Small cave, Petalas | EU887898 | EU887847 | EU887862 | MK993729 | EU887877 |
| *Dolichopoda pavesii* | Drogarati Cave, Sami, Kefalonia Island | EF217010 | EF216921 | EF216951 | MK993730 | EF216977 |
| *Dolichopoda giachinoi* | Megalospilio Cave, Monastirakion, Aitolo-Akarnania | EF217012 | EF216922 | EF216952 | MK993731 | EF216978 |
| *Dolichopoda kiriakii* | Kiriaki Cave, Korifè, Aghlia Kiriaki, Parga | EF217014 | EF216924 | EF216954 | MK993732 | EF216980 |
| *Dolichopoda lustriae* | Aghios Andreas Cave, Valtou M., Halkiopuli, Etolia | N/A | N/A | EU887863 | MK993733 | EU887878 |
| *Dolichopoda vandeli* | Cave over Kopais Lake, Orkomenos, Beotia | EF217039 | EF216932 | EF216962 | MK993734 | EF216992 |
| *Dolichopoda insignis* | Panos Cave, Marathon, Athene, Attica | EF217054 | EF216938 | EF216968 | MK993735 | EF217000 |
| *Dolichopoda petrochilosi* | Aghlia Joannis, Nea Pendeli, Athene, Attica | EF217053 | EF216937 | EF216967 | MK993736 | EF216999 |
| *Dolichopoda cassagnaui* | Aghlia Triada Cave, Karistos, Eubea Island | EF217035 | EF216931 | EF216961 | MK993737 | EF216991 |
| *Dolichopoda octhoniai* | Graspilea Cave, Ochtonia, Eubea Island | N/A | MK993667 | MK993655 | MK993738 | MK993661 |
| *Dolichopoda makrykapa* | Paralia Kilidau Cave, Lamari, Eubea Island | EF217041 | EF216941 | EF216971 | MK993739 | EF216993 |
| *Dolichopoda saraolacosi* | Abandoned mine, Atsitsa, Skyros isl. | N/A | MK993668 | MK993656 | MK993740 | MK993662 |
| *Dolichopoda naxia* | Zeus Cave, Filotas, Naxos Island, Cyklades | EU887909 | EU887853 | EU887868 | MK993741 | EU887882 |
| *Dolichopoda giulianae* | Moni Spilianis Cave, Pithagorion, Samos Island | EF217049 | EF216935 | EF216965 | MK993742 | EF216997 |
| *Dolichopoda calidnae* | Skalia cave, Skalia, Kalimnos Island, Dodecanese | EF217048 | EF216934 | EF216964 | MK993743 | EF216996 |
| *Dolichopoda matsakisi* | Ton Limnon Cave, Kastri, Kalavrita, Achaia | EF217022 | EF216927 | EF216957 | MK993744 | EF216985 |
| *Dolichopoda dalensi* | Kefalovrisi Cave, Argos, Argolide | EF217026 | EF216929 | EF216959 | MK993745 | EF216987 |
| *Dolichopoda unicolor* | River cave of Glyfada, Dirou, Aeropolis, Laconia | EF217045 | EF216940 | EF216970 | MK993746 | EF216994 |
| *Dolichopoda paraskevi* | Aghlia Paraskevi Cave, Skotinon, Iraklio | EF217030 | EF216942 | EF216972 | MK993747 | EF216988 |
| *Dolichopoda sbordonii* | Karain Cave, Antalya, Turkey | EF217050 | EF216936 | EF216966 | MK993748 | EF216998 |
| *Dolichopoda lyciae* | Gedelma cave, Antalya, Turkey | EU887918 | EU887855 | EU887870 | MK993749 | EU887884 |

| | | | | | | |
|---|---|---|---|---|---|---|
| *Dolichopoda noctivaga* | Coruh Valley, Erzurum, Turkey | EU887920 | EU887857 | EU887872 | MK993750 | EU887886 |
| *Dolichopoda euxina* | Vorontzovskaya, Caucasus, Russia | AY793622 | EF216945 | AY793566 | MK993751 | EF217002 |
| *Dolichopoda hyrcana* | Talysh cave, Lenkoran, Trancaucasia eastern | EU887919 | EU887856 | EU887871 | MK993752 | EU887885 |
| *Dolichopoda* sp. | N/A | N/A | EU887848 | N/A | N/A | N/A |

**Table S2.** Primer information and PCR conditions used in this study.

| Gene | Primer name | Sequence (5' – 30') | PCR condition | References |
|---|---|---|---|---|
| COI | COBL (F) | TYTCAACAAAYCAYAARGATATTGG | 94℃/1min, 5 X (94℃/30s, 45℃/40s, 72℃/60s), 35 X (94℃/30s, 51℃/40s, 72℃/60s), 72℃/10min | Huang et al. (2013) |
| | COBU (R) | TAAACTTCWGGRTGWCCAAARAATCA | | |
| 12S | 12S ai (F) | AAACTAGGATTAGATACCCTATTAT | 95℃/10min, 30 X (95℃/1min, 44℃/1min, 72℃/2min) | Simon et al. (1994) |
| | 12S bi (R) | AAGAGCGACGGGCGATGTGT | | |
| 16S | 16Sar (F) | CGCCTGTTTAACAAAAACAT | 95℃/10min, 30 X (95℃/1min, 50℃/1min, 72℃/2min) | Simon et al. (1994) |
| | 16Sbr (R) | CTCCGGTTTGAACTCA-GATCA | | |
| 18S | 18SF (F) | GACAACCTGGTTGATCCTGCCAGT | 95℃/5min, 35 X (94℃/45s, 49℃/45s, 72℃/1min) | Giribet et al. (1996) |
| | 18S4R (R) | GAATTACCGCGGCTGCTGG | | |
| 28S | 28S485 (F) | GACCCGTCTTGAAACACGA | 95℃/3min, 34 X (93℃/1min, 60℃/1min, 72℃/2min), 72℃/4min | Friedrich and Tautz (1997) |
| | 28S689 (R) | ACACACTCCTTAGCGGA | | |

**Table S3.** Dispersal multipliers for five time slices used in Biogeographical analyses.

0–30 Mya

| | A | B | C | D | E | F | G | H | I |
|---|---|---|---|---|---|---|---|---|---|
| A | 1 | 1 | 0.01 | 0.01 | 0.01 | 0.01 | 0.1 | 0.01 | 0.01 |
| B | 1 | 1 | 0.01 | 0.01 | 0.01 | 0.01 | 0.1 | 0.01 | 0.01 |
| C | 0.01 | 0.01 | 1 | 0.1 | 0.01 | 0.01 | 0.01 | 0.01 | 0.01 |
| D | 0.01 | 0.01 | 0.1 | 1 | 0.01 | 0.01 | 0.01 | 0.01 | 0.01 |
| E | 0.01 | 0.01 | 0.01 | 0.01 | 1 | 0.01 | 0.01 | 0.01 | 0.01 |
| F | 0.01 | 0.01 | 0.01 | 0.01 | 0.01 | 1 | 0.01 | 0.01 | 0.01 |
| G | 0.1 | 0.1 | 0.01 | 0.01 | 0.01 | 0.01 | 1 | 0.01 | 0.01 |
| H | 0.01 | 0.01 | 0.01 | 0.01 | 0.01 | 0.01 | 0.01 | 1 | 1 |
| I | 0.01 | 0.01 | 0.01 | 0.01 | 0.01 | 0.01 | 0.01 | 1 | 1 |

30–60 Mya

| | A | B | C | D | E | F | G | H | I |
|---|---|---|---|---|---|---|---|---|---|
| A | 1 | 1 | 0.01 | 0.01 | 0.01 | 0.01 | 0.1 | 0.1 | 1 |
| B | 1 | 1 | 0.01 | 0.01 | 0.01 | 0.01 | 0.1 | 0.1 | 0.1 |
| C | 0.01 | 0.01 | 1 | 0.1 | 0.01 | 0.01 | 0.01 | 0.01 | 0.01 |
| D | 0.01 | 0.01 | 0.1 | 1 | 0.01 | 0.01 | 0.01 | 0.01 | 0.01 |
| E | 0.01 | 0.01 | 0.01 | 0.01 | 1 | 0.01 | 0.01 | 0.01 | 0.01 |
| F | 0.01 | 0.01 | 0.01 | 0.01 | 0.01 | 1 | 0.01 | 0.01 | 0.01 |
| G | 0.1 | 0.1 | 0.01 | 0.01 | 0.01 | 0.01 | 1 | 0.1 | 0.1 |
| H | 0.1 | 0.1 | 0.01 | 0.01 | 0.01 | 0.01 | 0.1 | 1 | 1 |
| I | 1 | 0.1 | 0.01 | 0.01 | 0.01 | 0.01 | 0.1 | 1 | 1 |

60–90 Mya

| | A | B | C | D | E | F | G | H | I |
|---|---|---|---|---|---|---|---|---|---|
| 1 | 1 | 0.01 | 0.01 | 0.01 | 0.01 | 0.01 | 0.01 | 1 | 1 |
| 1 | 1 | 0.01 | 0.01 | 0.01 | 0.01 | 0.01 | 0.01 | 0.1 | 1 |
| 0.01 | 0.01 | 1 | 1 | 0.01 | 0.01 | 0.01 | 0.01 | 0.01 | 0.01 |
| 0.01 | 0.01 | 1 | 1 | 0.01 | 0.01 | 0.01 | 0.01 | 0.01 | 0.01 |
| 0.01 | 0.01 | 0.01 | 0.01 | 1 | 0.01 | 0.01 | 0.01 | 0.01 | 0.01 |
| 0.01 | 0.01 | 0.01 | 0.01 | 0.01 | 1 | 0.01 | 0.01 | 0.01 | 0.01 |
| 0.01 | 0.01 | 0.01 | 0.01 | 0.01 | 0.01 | 1 | 0.1 | 0.01 | 0.01 |
| 0.01 | 0.01 | 0.01 | 0.01 | 0.01 | 0.01 | 0.1 | 1 | 0.1 | 0.01 |
| 1 | 0.1 | 0.01 | 0.01 | 0.01 | 0.01 | 0.01 | 0.1 | 1 | 1 |

90–120 Mya

| | A | B | C | D | E | F | G | H | I |
|---|---|---|---|---|---|---|---|---|---|
| A | 1 | 1 | 0.01 | 0.01 | 0.01 | 0.01 | 0.01 | 0.01 | 0.1 |

| | | | | | | | | | |
|---|---|---|---|---|---|---|---|---|---|
| B | 1 | 1 | 0.01 | 0.01 | 0.01 | 0.01 | 0.01 | 0.01 | 0.01 |
| C | 0.01 | 0.01 | 1 | 1 | 0.1 | 0.1 | 0.01 | 0.01 | 0.01 |
| D | 0.01 | 0.01 | 1 | 1 | 0.1 | 0.1 | 0.01 | 0.01 | 0.01 |
| E | 0.01 | 0.01 | 0.1 | 0.1 | 1 | 0.1 | 0.01 | 0.01 | 0.01 |
| F | 0.01 | 0.01 | 0.1 | 0.1 | 0.1 | 1 | 0.01 | 0.01 | 0.01 |
| G | 0.01 | 0.01 | 0.01 | 0.01 | 0.01 | 0.01 | 1 | 0.1 | 0.01 |
| H | 0.01 | 0.01 | 0.01 | 0.01 | 0.01 | 0.01 | 0.1 | 1 | 1 |
| I | 0.1 | 0.01 | 0.01 | 0.01 | 0.01 | 0.01 | 0.01 | 1 | 1 |

120–150 Mya

| | A | B | C | D | E | F | G | H | I |
|---|---|---|---|---|---|---|---|---|---|
| A | 1 | 1 | 0.01 | 0.01 | 0.01 | 0.01 | 0.1 | 0.1 | 0.1 |
| B | 1 | 1 | 0.01 | 0.01 | 0.01 | 0.01 | 0.1 | 0.1 | 0.1 |
| C | 0.01 | 0.01 | 1 | 1 | 1 | 1 | 0.01 | 0.01 | 0.01 |
| D | 0.01 | 0.01 | 1 | 1 | 0.1 | 0.1 | 0.01 | 0.01 | 0.01 |
| E | 0.01 | 0.01 | 1 | 0.1 | 1 | 1 | 0.01 | 0.01 | 0.01 |
| F | 0.01 | 0.01 | 1 | 0.1 | 1 | 1 | 0.01 | 0.01 | 0.01 |
| G | 0.1 | 0.1 | 0.01 | 0.01 | 0.01 | 0.01 | 1 | 1 | 0.1 |
| H | 0.1 | 0.1 | 0.01 | 0.01 | 0.01 | 0.01 | 1 | 1 | 1 |
| I | 0.1 | 0.1 | 0.01 | 0.01 | 0.01 | 0.01 | 0.1 | 1 | 1 |

**Table S4.** Results of testing six biogeographic models in 'BioGeoBEARS.'

| Model | LnL | number of parameters | d | e | j | AIC | AIC weight |
|---|---|---|---|---|---|---|---|
| DEC | -51 | 2 | 0.0036 | 0.0004 | 0 | 106 | 0.0069 |
| DEC+J | -50.1 | 3 | 0.0017 | 1.0e-12 | 0.054 | 106.2 | 0.0063 |
| DIVALIKE | -48.5 | 2 | 0.0044 | 1.0e-12 | 0 | 101 | 0.084 |
| DIVALIKE+J | -45.92 | 3 | 0.0020 | 1.0e-12 | 0.050 | 97.84 | 0.41 |
| BAYAREALIKE | -48.5 | 2 | 0.0044 | 1.0e-12 | 0 | 101 | 0.084 |
| BAYAREALIKE+J | -45.92 | 3 | 0.0020 | 1.0e-12 | 0.050 | 97.84 | 0.41 |

**Table S5.** Results of testing six biogeographic models in 'BioGeoBEARS' in the unconstrained dating tree.

| Model | LnL | number of parameters | d | e | j | AIC | AIC weight |
|---|---|---|---|---|---|---|---|
| DEC | -62.03 | 2 | 0.0050 | 6.1e-05 | 0 | 128.1 | 0.0007 |
| DEC+J | -56.28 | 3 | 0.0018 | 1.0e-12 | 0.076 | 118.6 | 0.077 |
| DIVALIKE | -59.98 | 2 | 0.0069 | 0.0002 | 0 | 124 | 0.0052 |
| DIVALIKE+J | -54.5 | 3 | 0.0025 | 1.0e-12 | 0.076 | 115 | 0.46 |
| BAYAREALIKE | -59.98 | 2 | 0.0069 | 0.0002 | 0 | 124 | 0.0052 |
| BAYAREALIKE+J | -54.5 | 3 | 0.0025 | 1.0e-12 | 0.076 | 115 | 0.46 |

## B. Supplementary Figures

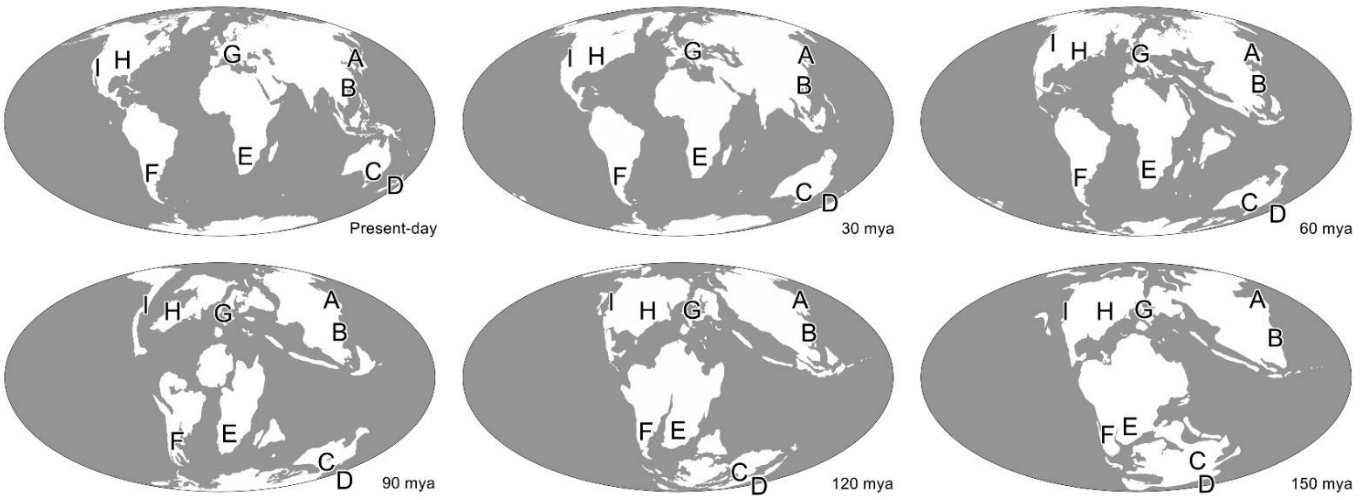


**Fig. S1.** Paleogeographic maps for 150 My using biogeographical analyses. (A) Eastern Asia; (B) Southeast Asia; (C) Tasmania, Australia; (D) New Zealand; (E) South Africa; (F) South America; (G) Mediterranean Region; (H) North America; (H) West Coast of North America; The maps were modified from Scotese (2021).

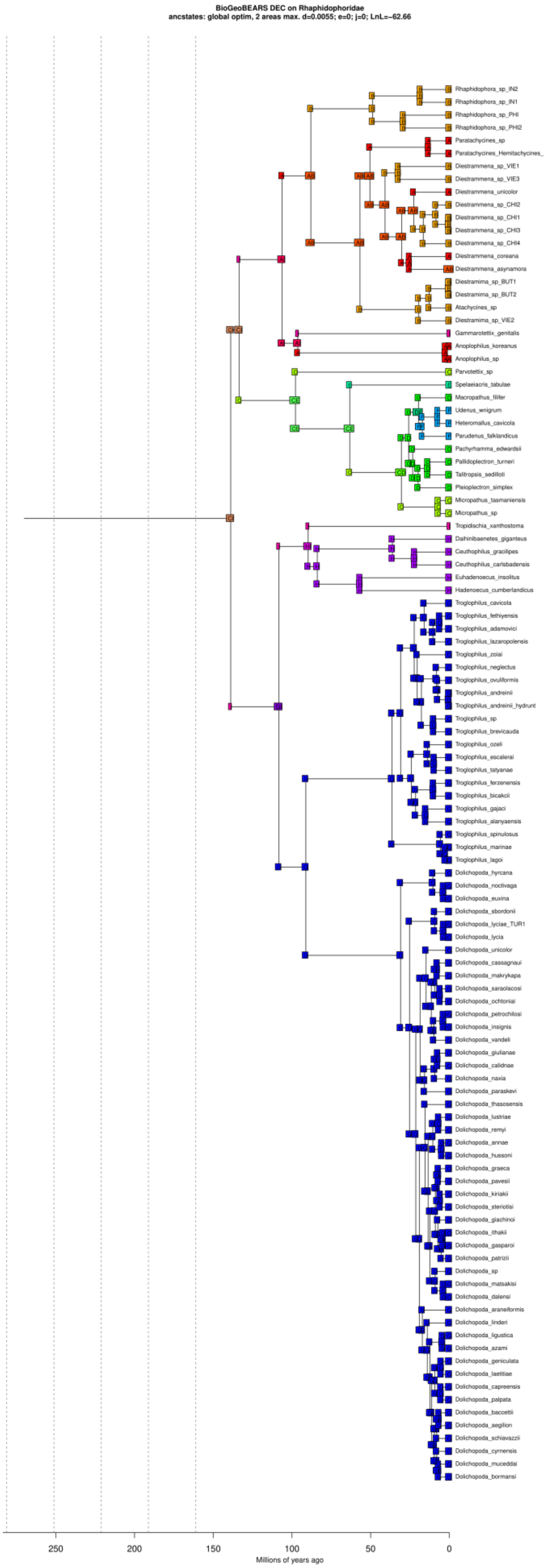


**Fig. S2.** Results of ancestral range estimation using the biogeographic model DEC in BioGeoBEARS (constraint tree).

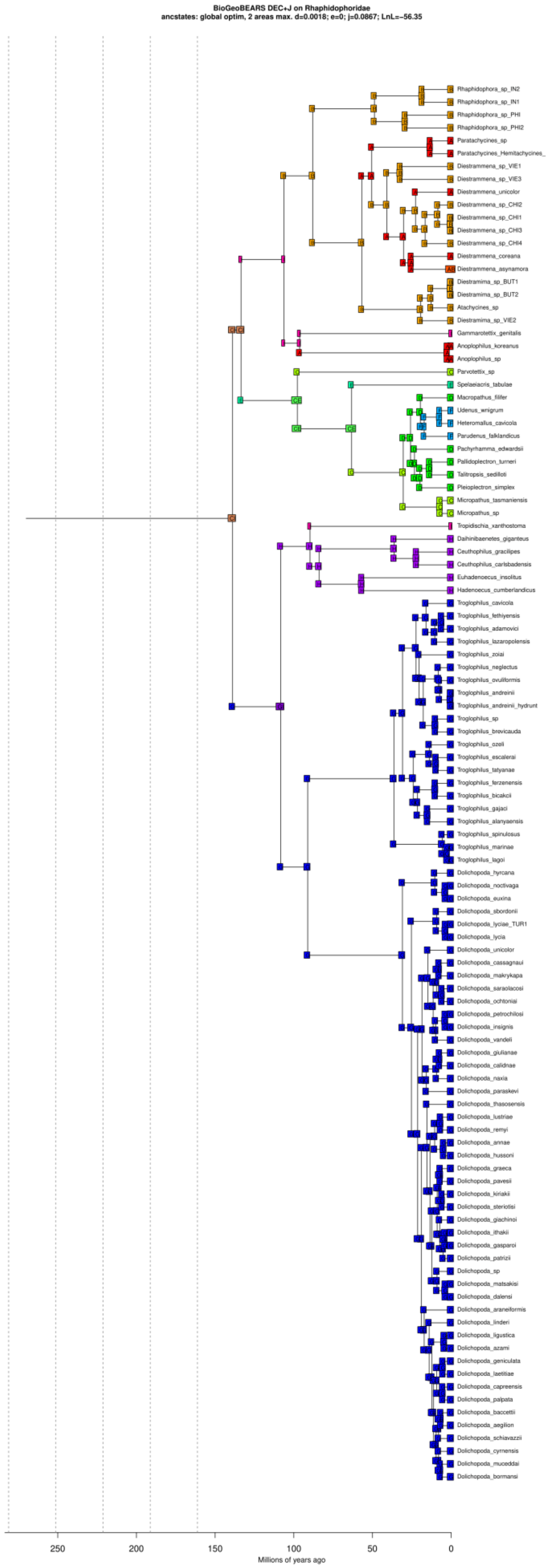


**Fig. S3.** Results of ancestral range estimation using the biogeographic model DEC+J in BioGeoBEARS (constraint tree).

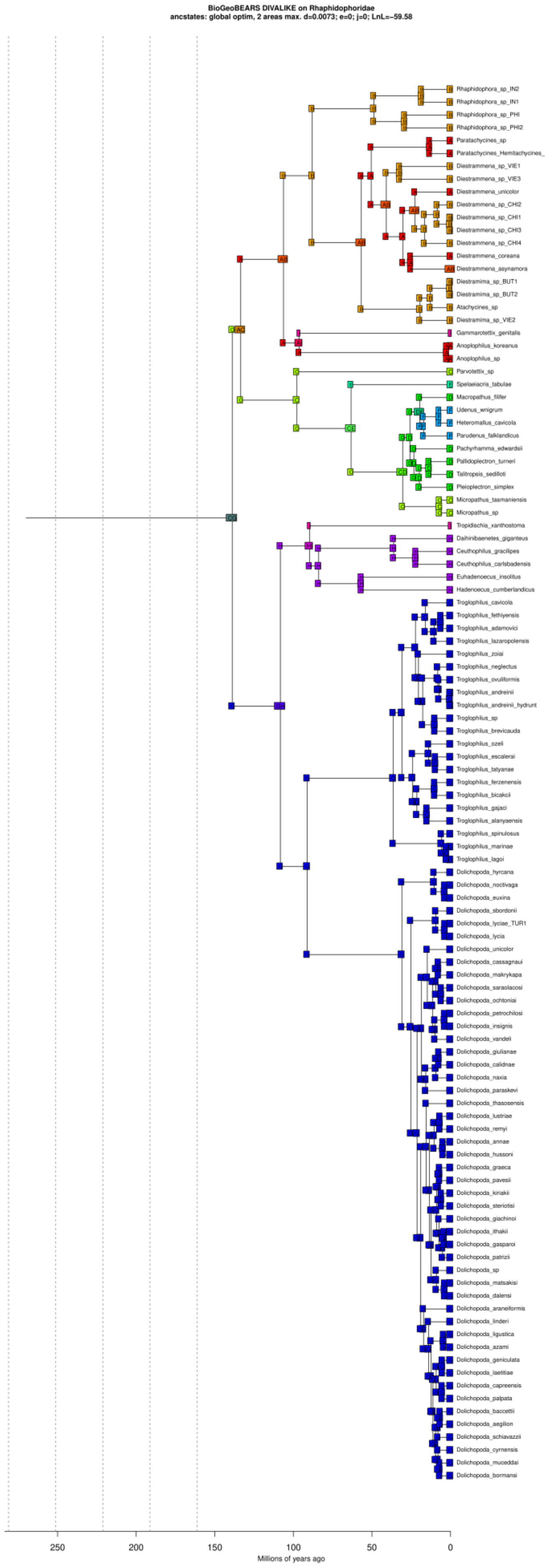


**Fig. S4.** Results of ancestral range estimation using the biogeographic model DIVALIKE in BioGeoBEARS (constraint tree).

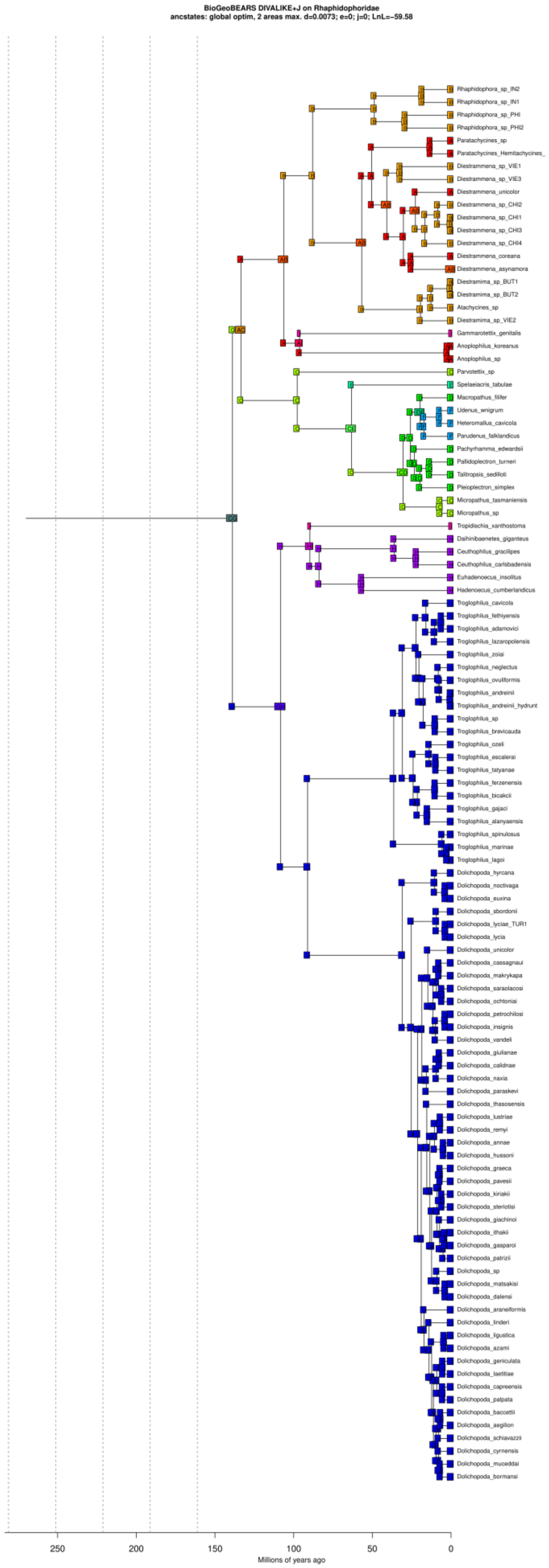


**Fig. S5.** Results of ancestral range estimation using the biogeographic model DIVALIKE+J in BioGeoBEARS (constraint tree).

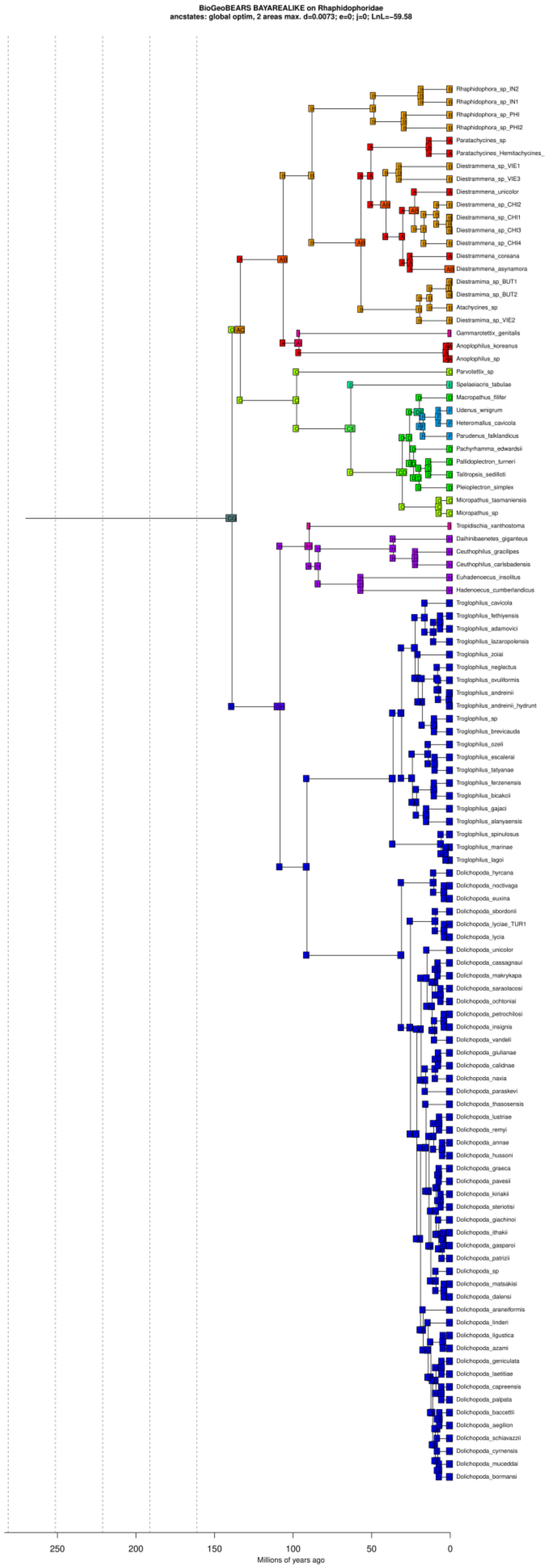


**Fig. S6.** Results of ancestral range estimation using the biogeographic model BAYAREALIKE in BioGeoBEARS (constraint tree).

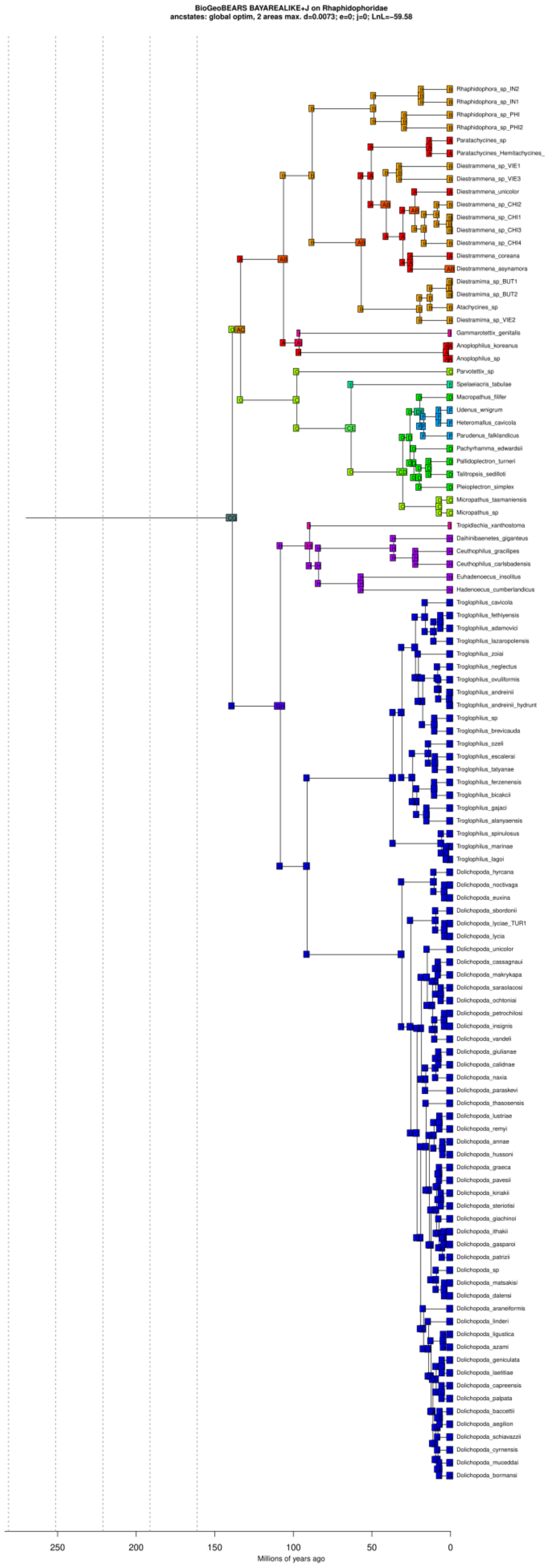


**Fig. S7.** Results of ancestral range estimation using the biogeographic model BAYAREALIKE+J in BioGeoBEARS (constraint tree).

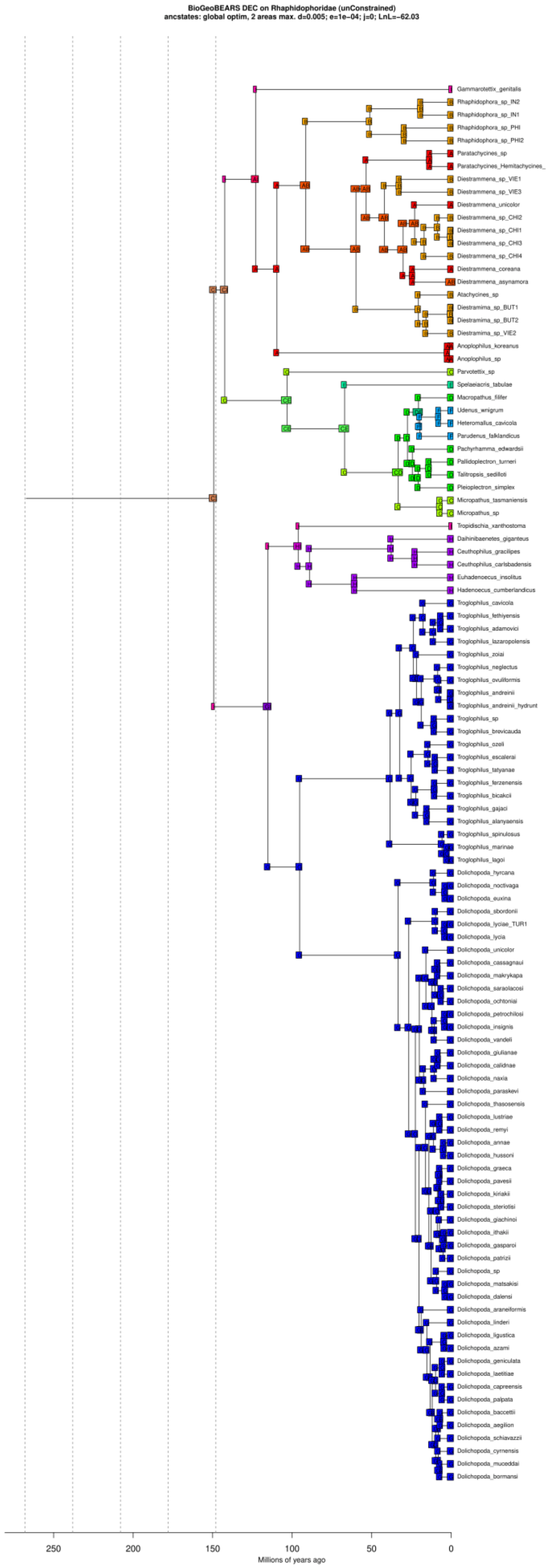


**Fig. S8.** Results of ancestral range estimation using the biogeographic model DEC in BioGeoBEARS (unconstraint tree).

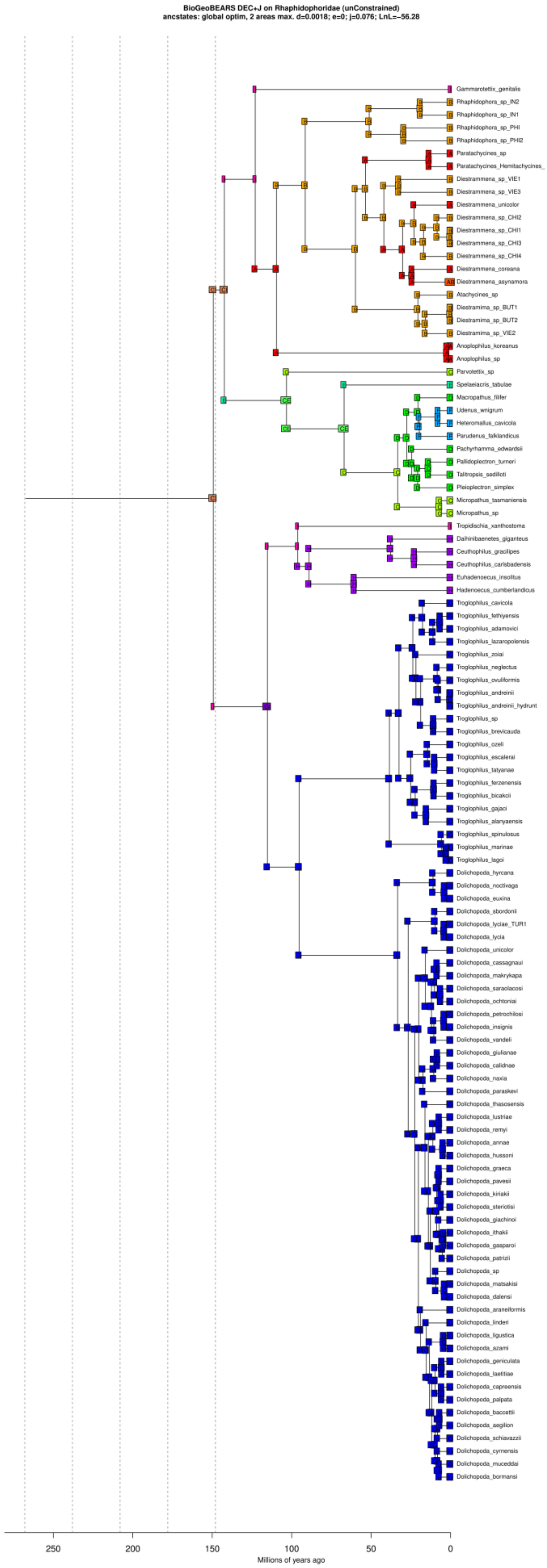


**Fig. S9.** Results of ancestral range estimation using the biogeographic model DEC+J in BioGeoBEARS (unconstraint tree).

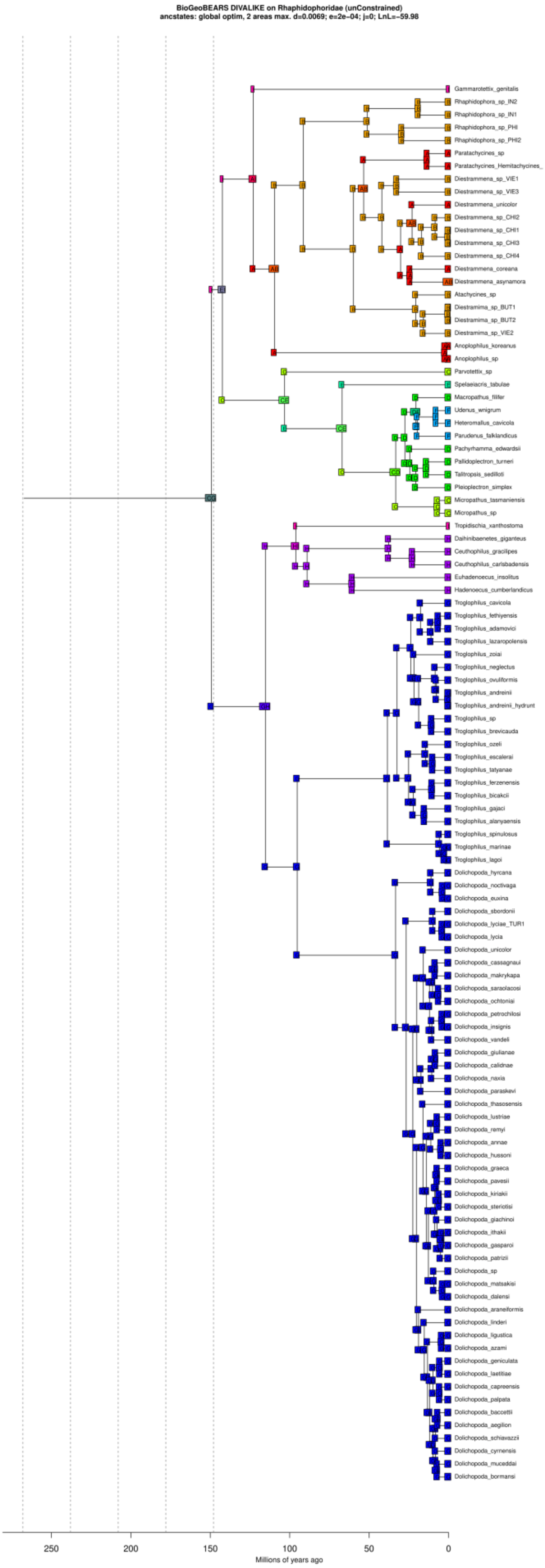


**Fig. S10.** Results of ancestral range estimation using the biogeographic model DIVALIKE in BioGeoBEARS (unconstraint tree).

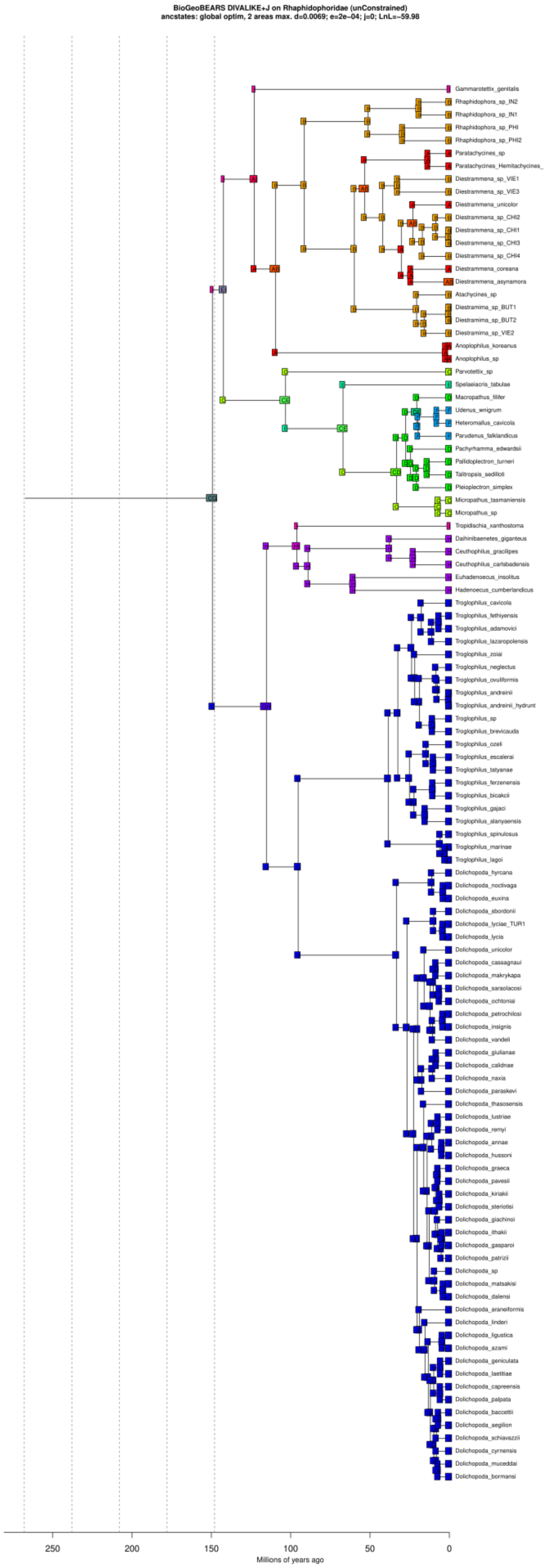


**Fig. S11.** Results of ancestral range estimation using the biogeographic model DIVALIKE+J in BioGeoBEARS (unconstraint tree).

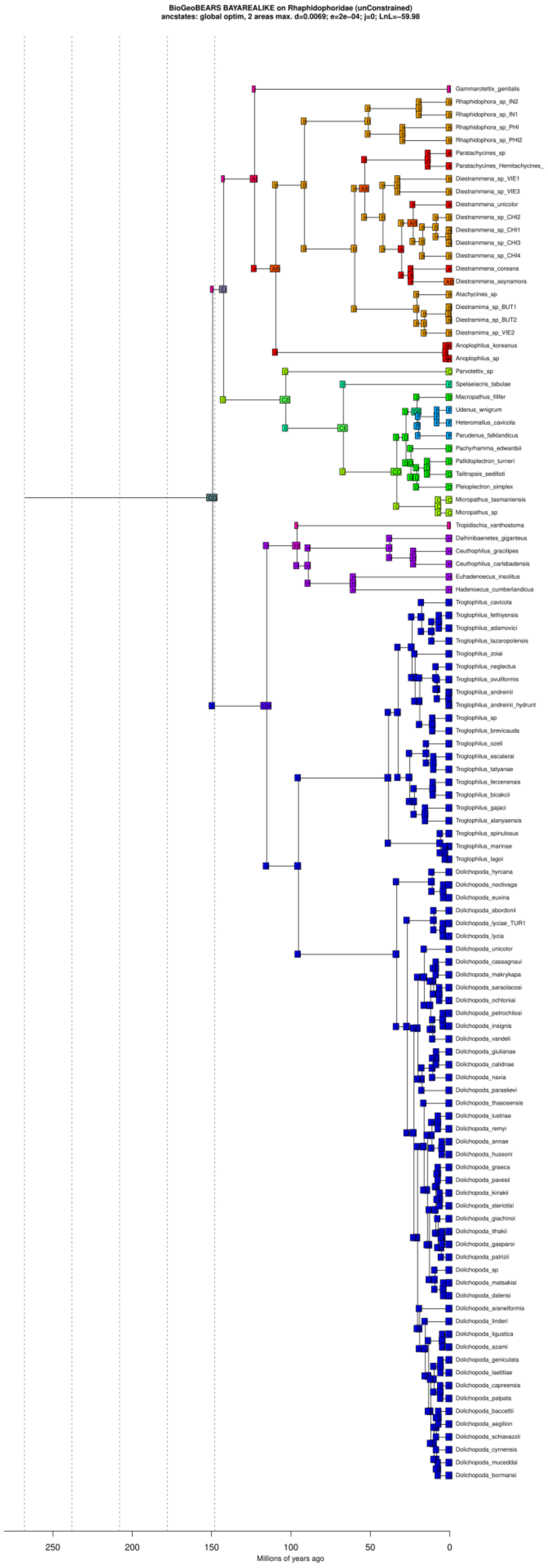


**Fig. S12.** Results of ancestral range estimation using the biogeographic model BAYAREALIKE in BioGeoBEARS (unconstraint tree).

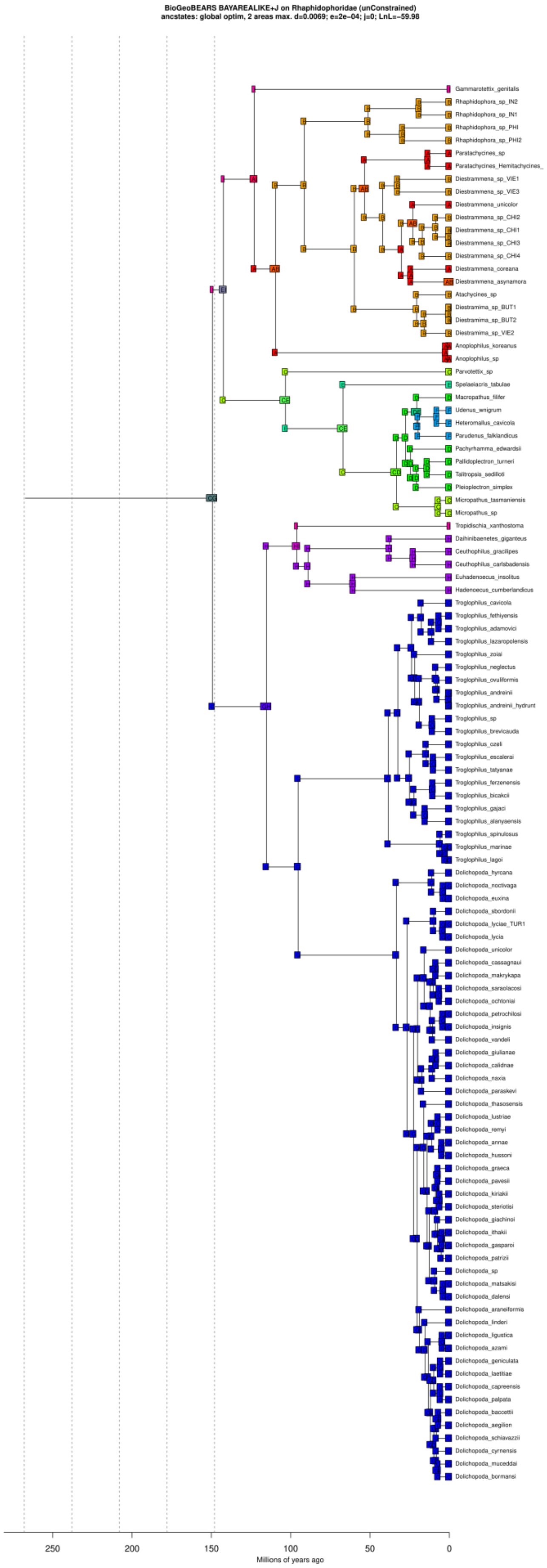


**Fig. S13.** Results of ancestral range estimation using the biogeographic model BAYAREALIKE+J in BioGeoBEARS (unconstraint tree).

## C. Supplementary Note

### Systematics

***Tachycines (Tachycines) coreana*** **(Yamasaki, 1969) stat. resurr.**

*Tachycines coreanus* Yamasaki, 1969: 616 (holotype - male, Korea: Jeju Is., Seong-gul Cave; in NSMT, Tokyo); Warchalowska-Sliwa & Kostia, 1993: 1; Otte, 2000: 56.

*Tachycines (Tachycines) coreanus* (Yamasaki): Storozhenko, 1990: 847; Jin & Xia, 1994: 18; Kostia, 1996: 109; Paik et al., 2010: 9, 66.

*Diestrammena coreana* (Yamasaki): Kwon & Huh, 1994: 48; Kim, 2013: 160.

*Diestrammena (Tachycines) coreana* (Yamasaki): Kim & Kim, 2002: 144; Storozhenko & Paik, 2007: 87; Storozhenko, 2008: 8; Paik et al., 2010: 36; Storozhenko et al, 2015: 97.

*Tachycines (Tachycines) coreana* (Yamasaki): Jung, 2001: 22.

*Tachycines (Tachycines) meditationis* Würmli, 1973: 1 (holotype - male, China; in NHMB, Basel), synonymized by Storozhenko (1990).

*Tachycines (Tachycines) asynamorus* Adelung, 1902: Qin et al., 2018: 453.

*Diesterammena apicalis* (nec Brunner-Wattenwyl): Kamijo, 1933: 49; Cho, 1959: 168; Cho, 1969: 765; Kwon & Huh, 1994: 48.

*Diagnostic description.* See Storozhenko et al., 2015

*Distribution.* Korea, China (Zhejiang, Jilin, Heilongjiang), Japan (Honshu, introduced to Hokkaido), and Russia (introduced to Vladivostok).

*Remarks.* Qin et al. (2018) considered *T. (T.) coreana* a synonym of *T. (T.) asynamorus* due to bristles on the hind metatarsus and the possibility of nymph (to quote the word in the paper, 'larva') and resurrected *T. (T.) meditationis* synthesized by Storozhenko (1990). However, the characteristics, bristles of the hind metatarsus in both species, which they suggested as the reason for the synonym, are insufficient. The two species are distinctly divided by male genitalia (see Storozhenko et al., 2015) and can be easily distinguished by the presence of stripe patterns on the hind legs that appear only in *T. (T.) asynamorus*. Therefore, in this study, *T. (T.) coreana* was resurrected, and the synonym of *T. (T.) meditationis* was restored.

## D. Supplementary References


1. Cho, P. (1959) A manual of the Orthoptera of Korea. *Human and Natural Science, Korea University*, **4**, 131–198. (In Korean)
2. Cho, P. (1969) *Illustrated Encyclopedia of Fauna and Flora of Korea, Vol. 10, Insecta (II)*, Samhwa Publishing Company, Seoul. (In Korean)
3. Friedrich, M., & Tautz, D. (1997) An episodic change of rDNA nucleotide substitution rate has occurred during the emergence of the insect order Diptera. *Molecular Biology and Evolution*, **14**, 644–653.
4. Giribet, G., Carranza, S., Baguna, J., Riutort, M., & Ribera, C. (1996) First molecular evidence for the existence of a Tardigrada+ Arthropoda clade. *Molecular biology and evolution*, **13**, 76–84.
5. Huang, J., Zhang, A., Mao, S., & Huang, Y. (2013) DNA barcoding and species boundary

delimitation of selected species of Chinese Acridoidea (Orthoptera: Caelifera). *PloS one*, **8**, e82400.

6. Jin, X.B. & Xia, K.L. (1994) An index-catalogue of Chinese Tettigoniodea (Orthopteroidea: Grylloptera). *Journal of Orthoptera Research*, **3**, 15–41.
7. Jung, S. (2001) *Studies on Orthoptera (Insecta) of Jeju Island, Korea*. PhD Thesis, Cheju National University, Jeju.
8. Kamijo, N. (1933) On a collection of insects from North Keisho-Do, Korea (II). *Journal of the Chosen Natural History Society*, **15**, 46–63. (In Japanese)
9. Kim, T. (2013) *Orthoptera of Korea*, p. 381. GEO Book, Seoul. (In Korean)
10. Kostia, D. (1996) The Aemodogryllinae (Orthoptera: Rhaphidophoridae) found in the Korean Peninsula. *Insecta Koreana*, **13**, 105–111.
11. Kwon, Y. & Huh, E. (1994) *Check List of Insects from Korea*. Kon-Kuk Univerisity Press, Seoul. (In Korean)
12. Scotese, C.R. (2021) An atlas of Phanerozoic paleogeographic maps: the seas come in and the seas go out. *Annual Review of Earth and Planetary Sciences*, **49**, 679–728.
13. Simon, C., Frati, F., Beckenbach, A., Crespi, B., Liu, H., & Flook, P. (1994) Evolution, weighting, and phylogenetic utility of mitochondrial gene sequences and a compilation of conserved polymerase chain reaction primers. *Annals of the entomological Society of America*, **87**, 651–701.
14. Storozhenko, S.Y. (1990) Review of the orthopteran subfamily Aemodogryllinae (Orthoptera, Rhaphidophoridae). *Entomologicheskoe Obozrenie*, **69**, 835–849. (In Russian)
15. Storozhenko, S.Y. (2008) *Diestrammena coreana* (Yamasaki, 1969) is newly recorded cave-cricket species (Orthoptera, Rhaphidophoridae) from Russia. *Far Eastern Entomologist*, **188**, 8.
16. Storozhenko, S.Y. & Paik, J. (2007) *Orthoptera of Korea*, p. 232. Dalnauka, Vladivostok.
17. Storozhenko, S.Y. & Paik, J. (2010). A new subfamily of the Rhaphidophoridae (Orthoptera: Stenopelmatoidea), with description of a new species of the genus *Anoplophilus* Karny, 1931 from Korea. *Zootaxa*, **2421**, 49–60.
18. Storozhenko, S.Y., Jeon, M. & Kim, T. (2015) *Monograph of Korean Orthoptera.* National Institute of Biological Resources, Incheon.
19. Otte, D. (2000) *Orthoptera Species File 8. Gryllacrididae, Stenopelmatidae, Cooloolidae, Schizodactylidae, Anastostomatidae and Rhaphidophoridae*. The Orthopterists' Society, Philadelphia.
20. Paik, J., Jung, S. & Storozhenko, S.Y. (2010) Some Orthopteran Insects (Orthoptera: Insecta) of Jeju Island (4). *Korean Journal of Soil Zoology*, **14**, 30–38.
21. Qin, Y., Wang, H., Liu, X. & Li, K. (2018) Divided the genus *Tachycines* adelung (orthoptera, rhaphidophoridae: Aemodogryllinae; aemodogryllini) from china. *Zootaxa,* **4374**, 451–475.
22. Warchalowska-Sliwa, E. & Kostia, D. (1993) Chromosomes of *Tachycines coreanus* Yamasaki, 1969 (Orthoptera: Rhaphidophoridae). Karyotype, C-bands and NOR's. *Folia Biologica*, **44**, 1–4.
23. Würmli, M. (1973) *Tachycines (Tachycines) meditationis* n. sp., eine neue Rhaphidophoridae (Saltatoria) aus China. *Mitteilungen der Entomologischen Gesellschaft Basel*, **23**, 1–9.
24. Yamasaki, T. (1969) Results of the speleological survey in South Korea 1966. XVII. Cave-dwelling camel crickets from South Korea. *Bulletin of the National Science Museum, Tokyo*, **12**, 615–621.
